# Surface Hopping Molecular Dynamics Simulations for Photochemistry Involving Pyrene and CH_3_Cl

**DOI:** 10.1021/acs.jpca.5c02583

**Published:** 2025-07-24

**Authors:** Elham Mazarei, Evgenii Titov, Peter Saalfrank

**Affiliations:** † Institut für Chemie, 426790 Universität Potsdam, Karl-Liebknecht-Str. 24-25, Potsdam-Golm D-14476, Germany; ‡ Institut für Physik und Astronomie, Universität Potsdam, Karl-Liebknecht-Str. 24-25, Potsdam-Golm D-14476, Germany

## Abstract

Pure or halogenated polycyclic aromatic hydrocarbons (PAHs) and saturated halogenated hydrocarbons are both classes of harmful chemicals found in Earth’s atmosphere, often involved in photochemical reactions. On a positive side, the photoreaction of PAHs with halogenated hydrocarbons serves as a model and offers routes to functionalized nanostructured carbon-based materials with tailored optoelectronic properties. Mechanistic studies on the photoreactions of these chemicals, possibly with each other, are therefore clearly of interest but still comparatively rare. In the present work, as a representative case study, the photophysics (spectra, excited-state lifetimes) and photoreaction dynamics of van der Waals or chemically bound complexes of pyrene (C_16_H_10_) and methyl chloride (CH_3_Cl) were investigated using a combination of computational techniques, thereby delivering time- and atom-resolved information on postexcitation processes. Structural optimizations of possible reactants and products, as well as excited states and absorption spectra, were obtained by semiempirical (AM1) configuration interaction singles (CIS) and (time-dependent) density functional theory (DFT), respectively. Nonadiabatic surface hopping dynamics (NASH) based on AM1-CIS provided excited-state lifetimes and were used to explore various photochemical channels of CH_3_Cl physisorbed or covalently bound to pyrene.

## Graphical abstract

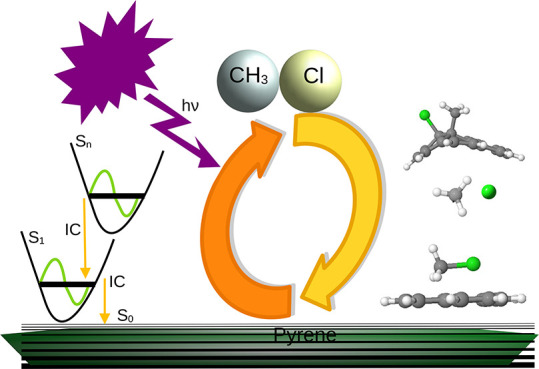

## 1 Introduction

Methyl chloride (CH_3_Cl) is the most abundant atmospheric halomethane and contributes to approximately 15% of the free chlorine radicals in the stratosphere.^1–3^ Understanding its properties and behavior is essential for developing strategies to mitigate ozone layer depletion.^4–6^

Spectroscopic studies of CH_3_Cl reveal important details about its electronic structure and photodissociation pathways.^4,7^ The lowest photodissociation channel of chlorofluorocarbons (CFCs) and halocarbons in general typically involves excitations from chlorine lone pairs (*n*) into C–Cl antibonding (σ*) orbitals induced by UVB or low UVC radiation.^4,8^ Photoinduced dissociation of CH_3_Cl was studied upon excitation with 193, 157, and 121 nm.^4^ At 193 or 157 nm, for example, mainly CH_3_ and Cl radicals are formed, while at even higher photon energies, C–H bonds can also be broken, and products such as CH_2_Cl, HCl, and CH_2_ emerge.^4–6,9^

Also, polycyclic hydrocarbons (PAHs), both natural and halogenated, are known to exist in the Earth’s atmosphere, acting in harmful ways, e.g., as carcinogens after returning to the Earth’s surface.^10^ On a positive side, graphene and PAHs, such as pyrene (C_16_H_10_), are of great interest for their fundamental electronic properties as prototypical two-dimensional (2D) materials with applications in nanoelectronics and sensing.^11,12^ There is significant interest in tuning the optical properties of these materials by functionalization, e.g., by (photo)­reactions with alkyl halides like CH_3_Cl.^12^ In that latter reference, properties of graphene (photo)­functionalized with CH_3_Cl were the focus, but no mechanistic study was provided. This paper will contribute to closing this gap, using pyrene as a molecular model for the extended 2D material.

Experimentally, the pyrene molecule allows for the addition of different functional groups through traditional synthetic techniques such as formylation/acetylation, halogenation, alkylation, oxidation, and borylation reactions, for example.^13,14^ Alternatively, functionalization can be done photochemically. Pyrene’s and its derivatives’ ultraviolet–visible (UV–vis) spectra have been thoroughly studied both experimentally^15^ and computationally.^16,17^ It displays three bands for pyrene in the lowest energy region. These bands correspond to transitions from the ground state *S*_0_ to the three singlet excited states *S*_2_, *S*_3_, and *S*_4_. The excitation wavelengths for these states are approximately 323, 265, and 232 nm, respectively.^15^ The lowest energy band (*S*_1_) is very weak.^17,18^

Photochemical functionalization of graphene using, e.g., Cl_2_ ^19^ or di-*tert*-butyl peroxide^20^ as reactants was demonstrated experimentally. However, to the best of our knowledge, there are no experimental works on the photoinduced functionalization of graphene/pyrene using CH_3_Cl. Our present study is motivated by the hypothesis that the photolysis of CH_3_Cl may serve as a source of reactive radicals to functionalize carbon-based 2D materials.

In this work, we explore the photoreactions of pyrene in contact with CH_3_Cl and analyze the resulting products, aiming to characterize elementary mechanisms. In particular, we examine both “physisorbed” (noncovalently bound) and “chemisorbed” complexes (covalently bound), compute their electronic absorption spectra in the ultraviolet–visible (UV–vis) region, and characterize excitations as molecular (CH_3_Cl) excited states (MS), charge transfer (CT), and hybrid (H) excited states. Further, we model nonadiabatic dynamics (using the nonadiabatic surface hopping (NASH) approach), considering both the excitation of a noncovalently bound pyrene–CH_3_Cl complex and the excitation of pyrene covalently modified with CH_3_ and Cl. In this way, possible photoreactions of CH_3_Cl with pyrene and possible back-photoreactions are studied, and time-resolved insight is also gained into the (nonreactive) photophysics of these systems. The paper is organized as follows. In the next section, Section 2, the methods adopted in this work will be summarized. Section 3 presents results from stationary quantum chemistry for ground (Section 3.1) and excited states (Section 3.2). Section 3.3 is devoted to nonadiabatic surface hopping dynamics. A final section concludes this work. Supporting Information gives further details on methods and results.

## 2 Models and Methods

### 2.1 Structures and Optimizations

We optimized the structures of pyrene and CH_3_Cl (see Figure 1), as well as different possible photoreactants and products, using DFT at the B3LYP+D3­(BJ)/ccpVDZ^21–25^ and ωB97X-D/cc-pVDZ levels^26^ and with the semiempirical AM1 (Austin Model 1) method^27^ with configuration interaction singles (CIS) based on floating occupation molecular orbitals (FOMOs).^28^ For details on the AM1/FOMO–CIS method, see refs 28 and 29. The width parameter was set to *w* = 0.1 hartree. CIS was used in view of subsequent excited-state calculations. In the AM1/FOMO–CIS method, an active space of 14 electrons in 13 frontier MOs was used for complexes of pyrene and CH_3_Cl. The HOMO–6, HOMO–5, and LUMO+5 of the physisorbed complex are located at CH_3_Cl. For the isolated CH_3_Cl molecule, the active space of 6 electrons in 4 orbitals was used. The HOMO–1, HOMO, and LUMO orbitals of the isolated molecule correspond to the HOMO–6, HOMO–5, and LUMO+5 orbitals of the physisorbed complex, respectively. The HOMO–2 of the isolated molecule was included to observe the intense absorption peak (HOMO–2 → LUMO) of the isolated CH_3_Cl. For pyrene, we chose the active space of 10 electrons in 10 orbitals—this corresponds to orbitals from HOMO–4 to LUMO+4, which are located on pyrene for the case of the physisorbed complex. We note that the physical picture of the CH_3_Cl dissociation along the C–Cl coordinate is described reasonably well (repulsive *S*_1_ state, reasonable *S*_0_ dissociation energy of 3.45 eV) using this method (see Figure S9). To better describe noncovalent (intermolecular) interactions in the case of the mainly van der Waals bound (vdW) pyrene–CH_3_Cl complex (see below), we incorporated vdW interaction terms, described by Lennard-Jones potentials, between pyrene and CH_3_Cl.^30^ The atomic vdW parameters were obtained from the optimized potentials for liquid simulations all-atom (OPLS-AA) force field.^31^ For atom pairs, the parameters were derived by calculating the geometric mean of the individual atomic parameters. Specific atomic values used are σ_C_ = 3.55 Å, σ_H_ = 2.42 Å, σ_Cl_ = 3.40 Å, ϵ_C_ = 0.07 kcal/mol, ϵ_H_ = 0.03 kcal/mol, and ϵ_Cl_ = 0.30 kcal/mol.

**Figure 1:**
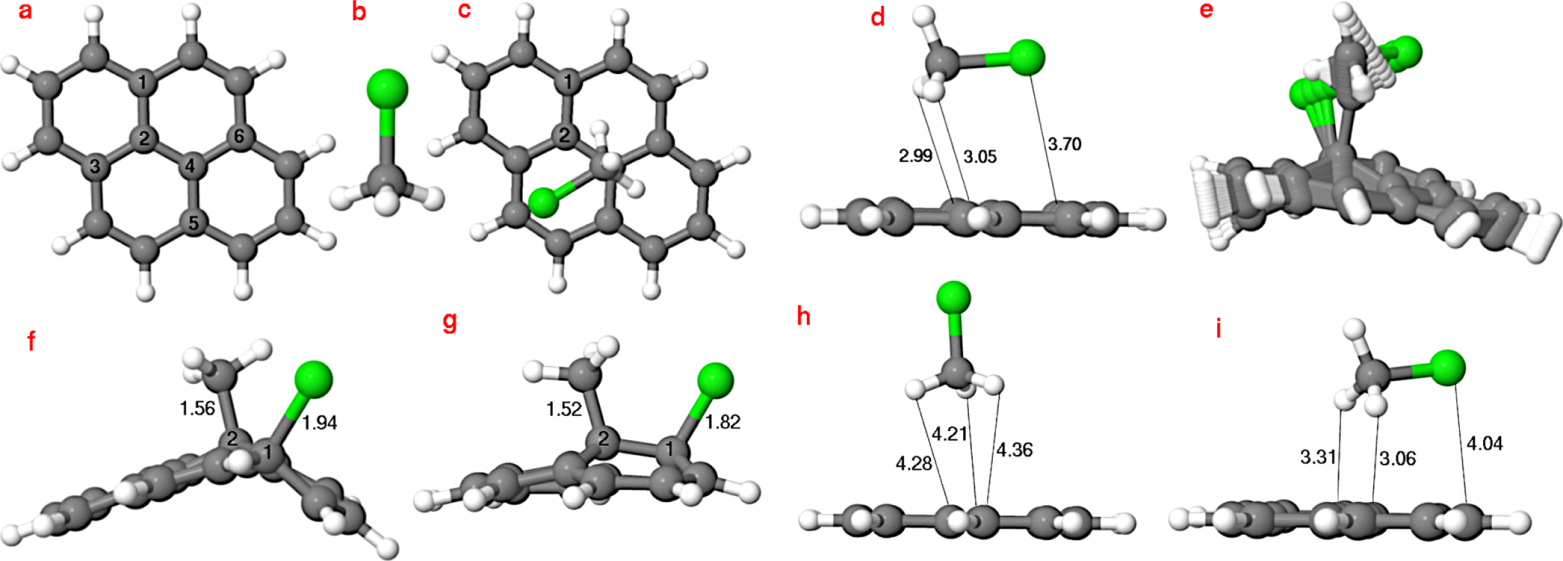
Optimized structures at the B3LYP+D3­(BJ)/cc-pVDZ level (a–f): panels (a,b) display the reactants [pyrene and CH_3_Cl, respectively], panels (c,d) show the top and side views of the noncovalently bound pyrene–CH_3_Cl complex, (e) the linear transit path (LTP), and (f) the side view of the pyrene–CH_3_Cl­(12-1Cl). Panel (g) is the optimized geometry obtained for pyrene–CH_3_Cl­(12-1Cl) at the AM1/FOMO–CIS level (with fixed H atoms of pyrene and without the LJ potential). Panel (h) is the optimized geometry for the pyrene–CH_3_Cl complex at the AM1/FOMO–CIS level without the LJ potential, and panel (i) is the optimized geometry for the pyrene–CH_3_Cl complex at the AM1/FOMO–CIS level with the LJ potential and with fixed H atoms of pyrene. H, C, and Cl are shown in white, gray, and green, respectively. Selected bond lengths and distances (Å) are also shown. The coordinates of the shown structures are provided in the Supporting Information.

All DFT calculations were performed using Gaussian 16,^32^ and the semiempirical calculations were done with MOPAC2002.^33^

### 2.2 Electronic Excited States and UV/Vis Spectra

Vertical absorption spectra for selected reactants and products were calculated using time-dependent DFT (TD-DFT)^34^ at the B3LYP/cc-pVDZ and ωB97X-D/cc-pVDZ levels, as well as the AM1/FOMO–CIS method. Only singlet excited states *S*_*i*_ after excitation from the ground state *S*_0_ were considered. The nature of the excited states was investigated using natural transition orbitals (NTO)^35^ and “fraction of transition density matrix” (FTDM) analysis.^36^ FTDM matrices were computed as^36,37^

1 $$F_{XY}=\frac{\underset{\mu \in X\;}{\sum }\underset{\nu \in Y}{\sum }{\left(S^{1/2}P^{\left[\mathrm{AO}\right]}S^{1/2}\right)}_{\mu \nu }^{2}}{\underset{\mu \in \mathrm{all}\;}{\sum }\underset{\nu \in \mathrm{all}}{\sum }{\left(S^{1/2}P^{\left[\mathrm{AO}\right]}S^{1/2}\right)}_{\mu \nu }^{2}}$$

Here, **P** ^[AO]^ is the transition density matrix (TDM) in the atomic orbital (AO) basis (computed with Multiwfn 3.8,^38,39^ ) and **S** is the AO overlap matrix (in the case of AM1/FOMO–CIS calculations, it was assumed that **S** = **I**, **I** being the identity matrix^40,41^ ).

The sums run over AOs belonging to fragments *X* or *Y*, or the entire system (“all”). The diagonal elements *F*_*XX*_ represent local excitations (LE) (localized on fragment *X*), while the off-diagonal elements *F*_*XY*_, *Y* ≠ *X* correspond to charge transfer (CT) excitations (from fragment *X* to fragment *Y*). In the following, we will present the FTDM elements as percentages, denoted as *F*_*XY*_ × 100%.

Vertical UV/vis spectra were calculated with TD-DFT or AM1-CIS, as

2 $$I\left(E\right)=\overset{N_{\mathrm{states}}}{\underset{i=1}{\sum }}f_{i\;}g\left(E-E_{i};\;\gamma \right)$$

where *g*(*E* – *E*_*i*_; γ) are Gaussians $g=\exp\left(-\frac{E-E_{i}}{2\gamma^{2}}\right)$ centered at *S*_0_ → *S*_*i*_ excitation energy *E*_*i*_ and with a width parameter γ. In the vertical case, the initial geometries were optimized ones. Further, *f*_*i*_ is the computed oscillator strength for the transition from the ground state to the excited state *i*, and *N*_states_ is the number of considered states. Alternatively, spectra were calculated from sampling initial geometries from a Langevin ground-state trajectory run at *T* = 300 K with AM1/FOMO–CIS, with vdW corrections for the physisorbed complex. The Langevin trajectories were 20 ps long with a time step of 0.2 fs. The friction coefficients for nonfixed atoms (see below) were set to 4.3 × 10^12^ s^–1^. For each system, geometries were sampled every 20 fs, starting from 500 fs, giving 100 initial configurations. Excitation energies and oscillator strengths were then calculated at each sampling point, and spectra were derived from eq 2, after replacing the sum over states by a double sum over states *i* and geometries α, *E_i_,* and *f*_*i*_ by *E*_*i*,α_ and *f*_*i*,α_, and dividing by *N*_samp_, the number of sampling points. If not stated otherwise, in all calculations below, a broadening factor γ = 0.18598 eV (which corresponds to 1500 cm^–1^) has been used.

### 2.3 Nonadiabatic Molecular Dynamics (NAMD) Simulations

Ground-state Langevin MD trajectories at *T* = 300 K were also used to provide initial geometries and velocities for nonadiabatic SH simulations, using AM1/FOMO–CIS and the same sampling scheme as above. The latter were performed with Tully’s fewest switching approach,^42^ coupled with AM1/FOMO–CIS.^29^ SH trajectories were propagated for 10 ps with a time step of 0.1 fs. The nuclei were propagated using classical mechanics on adiabatic potential energy surfaces (PESs), including the 12 lowest singlet states (*S*_0_ to *S*_11_). Which of these states was selected as the initial state will be described below. The time-dependent electronic wave function was propagated using a local diabatization method,^29,43^ which can properly account for trivial crossings.^43,44^ To address the issue of overcoherence in the original surface hopping algorithm, an energy-based decoherence correction was applied.^45^

When considering the pyrene/CH_3_Cl systems for photochemistry (see below), the hydrogen atoms of pyrene were frozen at their ground-state equilibrium in-plane position in all MD simulations to mimic interaction with a planar graphene surface. The surface hopping (and the Langevin dynamics) calculations were performed with the MOPAC-PI program.^46^

## 3 Results and Discussion

### 3.1 Ground State Structures and Energies

Figure 1a shows the structure of pyrene optimized at the B3LYP+D3 level of theory. When a single CH_3_Cl (Figure 1b) is added, we find the most stable arrangement considered here to be a “physisorbed”, noncovalently bound structure in which CH_3_Cl remains largely intact and is arranged as shown in Figure 1c (top view) and d (side view). We call this structure “pyrene–CH_3_Cl complex” from now on. We also considered “chemisorbed”, covalently bound species in which CH_3_Cl was dissociated and Cl added to one of the inner C atoms of pyrene (labeled C1 in the figure) and CH_3_ on a nearest-neighbor inner C atom, C2. We call this structure, shown in Figure 1f, “pyrene–CH_3_Cl­(12-1Cl)” from now on. Figure 1e presents the linear transit path (LTP) for a hypothetical ground-state reaction, starting from the noncovalent pyrene–CH_3_Cl complex (Figure 1c,d) and transitioning to pyrene–CH_3_Cl­(12-1Cl) (Figure 1f). Finally, Figure 1g–i shows the optimized geometries of “chemisorbed” and “physisorbed” complexes obtained at the AM1/FOMO–CIS level, with and without van der Waals corrections included. The latter proves important to reproduce the parallel (as opposed to upright) orientation of physisorbed CH_3_Cl on pyrene–in agreement with B3LYP+D3.

There are many (other) possibilities to add CH_3_ and Cl to pyrene, either on one side (as shown) or on opposite sides^12^ of pyrene, at closest neighboring C atoms (e.g., 12 or 24 in Figure 1), as well as at second-nearest and third-nearest neighbors. Additionally, the energy slightly varies depending on whether the group is added to a carbon atom near the cluster’s edge or on the “surface” (this means that 12-1CH_3_ and 12-1Cl have different energies). There is also the possibility that Cl and CH_3_ add to “outer” C atoms with H atoms, or the possibility to replace the H atoms of pyrene by Cl or CH_3_. In the current work, in order to mimic CH_3_Cl adsorption on one side of graphene (for which pyrene is a molecular model), we only considered covalently bound species with Cl and CH_3_ on one side of pyrene, adsorbing at inner C atoms, but allowed for different positions.

Table 1 displays the adsorption energies (*E*_ads_) for dissociated CH_3_Cl on top of pyrene with CH_3_ and Cl on nearest-neighbor C atoms, calculated with B3LYP-D3­(BJ)/cc-pVDZ, ωB97X-D3/cc-pVDZ, and AM1/FOMO–CIS methods. We used the “physisorbed” pyrene–CH_3_Cl complex as a reference to calculate the energy of the products, i.e., *E*_ads_ = *E*(product) – *E*(pyrene–CH_3_Cl). [Note that in calculating *E*_ads_, we did not account for the energy required to generate the CH_3_ and Cl radicals before their attachment to pyrene. We note that the homolytic dissociation energy of CH_3_Cl is approximately 3.7 eV, according to high-level ab initio calculations.^4^] We further mention that the energy difference between the physisorbed pyrene–CH_3_Cl complex and the reactants (free pyrene and CH_3_Cl) is −0.23 and −0.24 eV at the levels of ωB97X-D3/cc-pVDZ and B3LYP-D3­(BJ)/cc-pVDZ, respectively. At the AM1/FOMO–CIS level, it is −0.31 eV (without vdW corrections) and −0.40 eV (with vdW corrections—no matter if H atoms are fixed in pyrene or reoptimized), respectively.

**Table 1:** Adsorption Energies (*E*_ads_ in eV) for CH_3_Cl on Top of Pyrene at Different Nearest-Neighbor Positions Obtained Using B3LYP-D3­(BJ)/cc-pVDZ, ωB97X-D/cc-pVDZ, and AM1/FOMO–CIS Methods^a^

	B3LYP-D3(BJ)/cc-pVDZ	ωB97X-D/cc-pVDZ	AM1/FOMO–CIS
Pyrene–CH_3_Cl	0.0	0.0	0.0
12-1CH_3_	+2.45	+2.31	+2.21
12-1Cl	+2.41	+2.26	+2.19
24	+2.84	+3.12	+3.04

a *E*_ads_ values at the AM1/FOMO–CIS level are calculated without considering the LJ potential.

According to these findings and Table 1, the physisorption mode (pyrene–CH_3_Cl complex) is indeed the most stable product for the reaction between pyrene and CH_3_Cl (Figure 1d). Among the nearest-neighbor chemisorbed species (12-1CH_3_, 12-1Cl, and 24), pyrene–CH_3_Cl­(12-1Cl) is the most stable. Besides the physisorbed species, we chose only the pyrene–CH_3_Cl­(12-1Cl) system for further investigations below.

### 3.2 Excited States and Spectra

Further, we investigated the electronically excited states of possible reactants and products of photoreactions, namely CH_3_Cl, pyrene, the pyrene–CH_3_Cl complex, and pyrene–CH_3_Cl­(12-1Cl) using AM1/FOMO–CIS, which is later also used for (NASH) dynamics simulations. To assess the performance of the semiempirical method, we performed additional calculations with TD-DFT, namely TD-B3LYP+D3­(BJ)/cc-pVDZ and TD-ωB97X-D/cc-pVDZ. Some results of these latter calculations, if not already shown here, are presented in Section S1. For AM1/FOMO–CIS, the lowest excited 42 singlet states were computed; for TD-DFT, the lowest 50 excited states.

Figure 2 presents vertical, broadened absorption spectra for (a) CH_3_Cl, (b) pyrene, (c) the pyrene–CH_3_Cl complex, and (d) the pyrene–CH_3_Cl­(12-1Cl) system, obtained using AM1/FOMO–CIS and eq 2. As mentioned, the active space for pyrene–CH_3_Cl was chosen to be 7 occupied and 6 virtual orbitals. Based on inspection, we set the active space for CH_3_Cl as 3 occupied and 1 virtual orbitals, and for pyrene, 5 occupied and 5 virtual orbitals.

**Figure 2:**
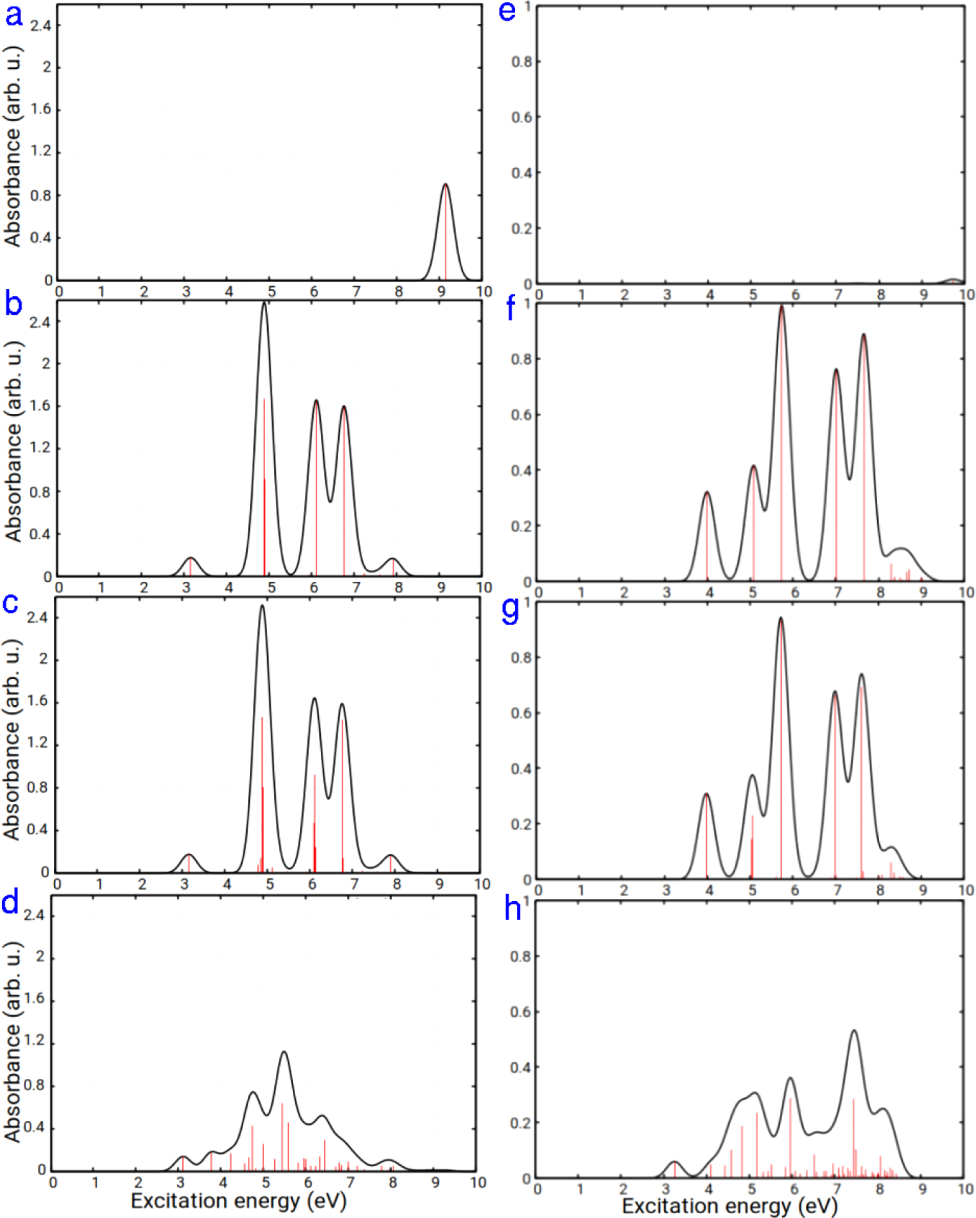
Absorption spectra for (a) CH_3_Cl, (b) pyrene, (c) pyrene–CH_3_Cl complex, and (d) pyrene–CH_3_Cl­(12-1Cl) system at the AM1/FOMO–CIS level, and for (e) CH_3_Cl, (f) pyrene, (g) pyrene–CH_3_Cl complex, and (h) pyrene–CH_3_Cl­(12-1Cl) system at the TD-ωB97X-D/cc-pVDZ level.

For the CH_3_Cl molecule, two bands are observed: one at 9.14 eV and another at 4.81 eV. The intensity of the latter band is very low and invisible on the scale of Figure 2a. Eden et al.^3^ investigated the vacuum ultraviolet (VUV) photoabsorption spectra of CH_3_Cl and identified the lowest-energy weak absorption band around 7.27 eV, which is blue-shifted with respect to the *S*_1_ excitation energy calculated with AM1/FOMO–CIS, and observed strong peaks starting at ∼8.8 eV, which is slightly red-shifted compared to our *S*_2_ calculated energy.

The pyrene spectrum exhibits a main maximum at 4.88 eV (with two prominent transitions at 4.87 and 4.89 eV) and four other peaks around 3.1 eV (weak), 6.1 and 6.8 eV (strong), and close to 8 eV (Figure 2b). The experimental vertical absorption energies for pyrene are 3.82 (3.84) and 3.56 (3.36) eV.^47,48^ The latter is slightly blue-shifted in comparison to the calculated *S*_1_ excitation energy. The pyrene–CH_3_Cl spectrum (Figure 2c) shows a very similar profile. (The ∼9 eV peak of CH_3_Cl is not seen in Figure 2c because not enough states were computed.) The only slight differences between the two spectra suggest a weak interaction between pyrene and CH_3_Cl in the pyrene–CH_3_Cl complex. However, the spectrum of the pyrene–CH_3_Cl­(12-1Cl) system differs considerably from that of the physisorbed complex, reflecting strong interactions between pyrene and the covalently attached CH_3_ and Cl ligands. For example, the spectrum exhibits a maximum intensity at 5.49 eV, while the pyrene–CH_3_Cl complex shows a maximum intensity at 4.88 eV, according to the AM1/FOMO–CIS method. Also, the intensities are quite different.

Figure 2 also displays the absorption spectra for (e) CH_3_Cl, (f) pyrene, (g) the pyrene–CH_3_Cl complex, and (h) the pyrene–CH_3_Cl­(12-1Cl) system, calculated at the TD-ωB97X-D/cc-pVDZ level of theory for the first 50 excited states. The first (degenerate) band for CH_3_Cl is located at 7.51 eV, but its intensity is very low. The second (also degenerate) band, at 9.74 eV, is slightly visible in the spectrum (Figure 2e). The pyrene, pyrene–CH_3_Cl complex, and pyrene–CH_3_Cl­(12-1Cl) systems show maximum intensities at 5.74, 5.73, and 7.45 eV, respectively, at the ωB97X-D/cc-pVDZ level. Comparing Figure 2a–d with Figure 2e–h reveals a red shift in the spectrum for the AM1/FOMO–CIS method. Again, we see that the ωB97X-D/cc-pVDZ level yields similar spectra for pyrene and the pyrene–CH_3_Cl complex.

We now analyze the nature of these excited states for the physisorbed and chemisorbed CH_3_Cl/pyrene systems. In particular, we are interested in excited states localized at CH_3_Cl, which we call in what follows the “molecular states” (MS), and in charge transfer (CT) between CH_3_Cl (M) and pyrene (P). Figure 3a,b displays MOs, representing two MS excited states for the pyrene–CH_3_Cl complex, calculated with AM1/FOMO–CIS. Both situations describe excitations from *n* to (C–Cl) σ* orbitals, similar to what one finds for free CH_3_–Cl, corresponding to transitions from *S*_0_ to *S*_7_ (in Figure 3a) or to *S*_9_ (Figure 3b), now for the physisorbed molecules. Both excitations are expected to lead to a weakening/breaking of the C–Cl bond. The corresponding transition energies (oscillator strengths) are 4.83 eV (0.14) and 4.88 eV (0.03), respectively.

**Figure 3:**
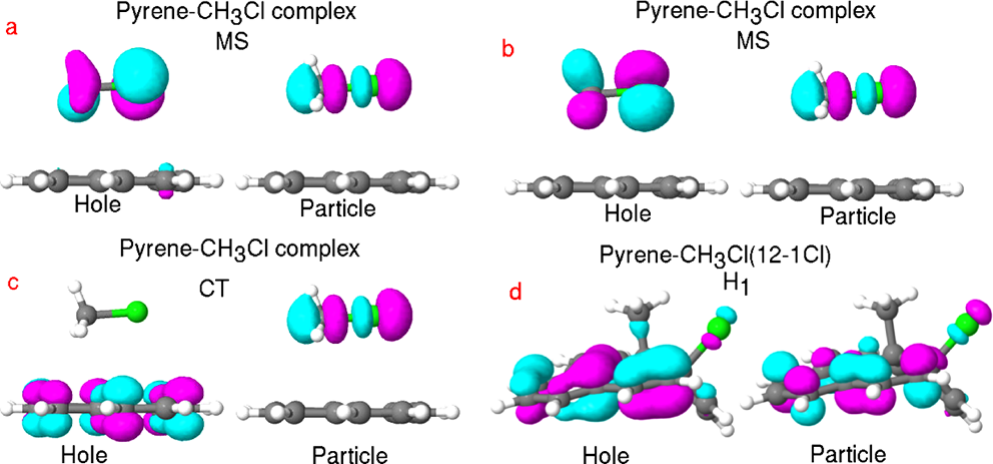
Hole and particle (represented with dominant conventional molecular orbital pairs) obtained at the AM1/FOMO–CIS level: panels (a,b) display MSs (*S*_7_ and *S*_9_), panel (c) displays the CT state (*S*_19_) for pyrene–CH_3_Cl, and panel (d) displays the hybrid state H_1_ (*S*_4_) for pyrene–CH_3_Cl­(12-1Cl).

Figure 3c displays the hole and particle MOs of a CT excited state for the (physisorbed) pyrene–CH_3_Cl complex, with negative charge flowing from pyrene to CH_3_Cl, into a C–Cl antibonding orbital, again calculated with AM1/FOMO–CIS. This CT state corresponds to an *S*_0_ → *S*_19_ transition, with an excitation energy of 6.49 eV. The oscillator strength for this transition is zero, however, i.e., it cannot be directly excited optically.

Figure 3d displays the hole and particle MOs of a hybrid excited state (H_1_) for (chemisorbed) pyrene–CH_3_Cl­(12-1Cl) at the AM1/FOMO–CIS level. For the chemisorbed species, no clear pure M or P local excitations or CT states are seen. Rather, both MOs show contributions from pyrene’s π-system and the CH_3_ and Cl orbitals. Closer inspection shows 21% M → P and 50% P → P contributions for the H_1_ transition, corresponding to an excitation from *S*_0_ to *S*_4_ with an energy (oscillator strength) of 4.23 eV (0.17) (see FTDM analysis below).

We also analyzed NTOs representing MS and CT excited states for the pyrene–CH_3_Cl complex and pyrene–CH_3_Cl­(12-1Cl) at the B3LYP+D3­(BJ)/cc-pVDZ level for comparison (see Figure S1). Now, the lowest MS state of the noncovalent pyrene–CH_3_Cl complex is found to be *S*_43_ (7.30 eV) when using B3LYP and *S*_26_ (7.50 eV) when using ωB97X-D. Recall that at the AM1/FOMO–CIS level, the lowest MS states for pyrene–CH_3_Cl complex are found to be *S*_7_ (4.83 eV) and *S*_9_ (4.88 eV). Clearly, there is a disagreement in state ordering (and absolute energies) when comparing AM1/FOMO–CIS and TD-DFT results, despite the overall spectra look similar. For surface hopping calculations, however, we decided to use the semiempirical level, considering the prohibitive computational cost of TD-DFT NAMD simulations with several dozen electronic states.

Figure 4 illustrates FTDM matrices for the lowest 42 singlet excited states *S*_*i*_ of the (physisorbed) pyrene–CH_3_Cl complex, calculated with AM1/FOMO–CIS. Each state is represented by a 2 × 2 matrix, with its off-diagonal elements *F*_PM_ and *F*_MP_ representing CT contributions (from P to M, upper right, and M to P, lower left), and the diagonal elements correspond to either pyrene (P to P) or molecular (M to M) LE contributions. Excitation energies and oscillator strengths are also indicated.

**Figure 4:**
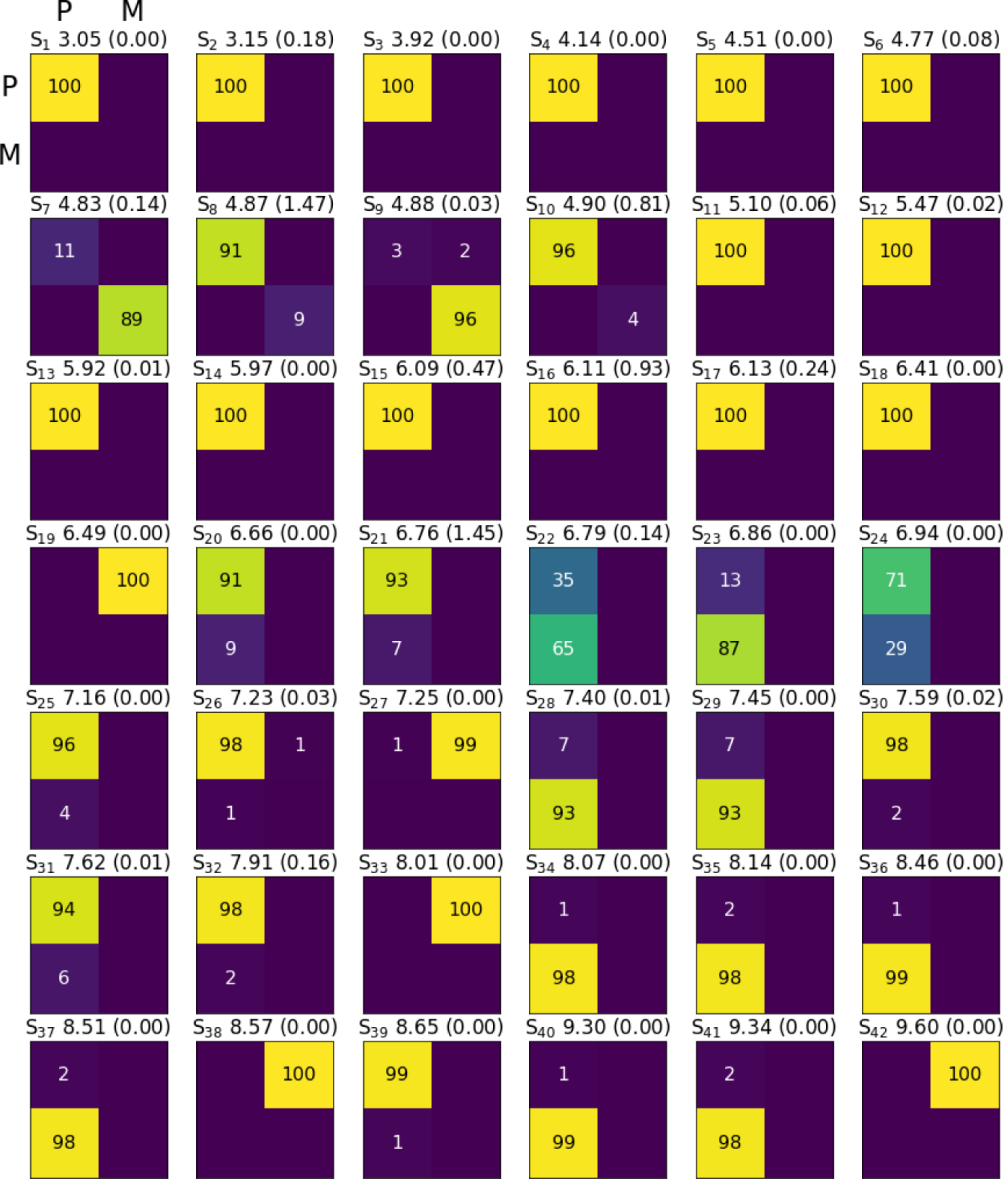
FTDM matrices for the first 42 excited states of the (physisorption) pyrene–CH_3_Cl complex at the AM1/FOMO–CIS level. P and M letters refer to the pyrene surface and CH_3_Cl molecule, respectively. *S*_1_ to *S*_42_ refer to excited state numbers from 1 to 42, the value after the state number is the transition energy in eV, and the value in parentheses is the oscillator strength of the transition from the ground state.

We see that states *S*_1_ to *S*_6_ are pure pyrene LEs for the pyrene–CH_3_Cl complex, with some of them being dark. States *S*_7_ and *S*_9_ (at 4.83 and 4.88 eV, respectively) are the already mentioned MSs, and state *S*_19_ (6.49 eV) is the dark CT state (with electron transfer from P to M) as shown in Figure 3c.

From Figure 5, where FTDMs for the (chemisorbed) pyrene–CH_3_Cl­(12-1Cl) are shown, we see that the lowest hybrid states are *S*_4_ (4.23 eV, “H_1_” in Figure 3d) and *S*_5_ (4.57 eV, “H_2_”) at the AM1/FOMO–CIS level. We see that in general, for pyrene–CH_3_Cl­(12-1Cl), all excited states are hybrid states, and no clear MS and CT classifications are possible as mentioned. Also, due to the lower symmetry, most transitions have nonvanishing oscillator strengths, in contrast to the (physisorbed) pyrene–CH_3_Cl complex.

**Figure 5:**
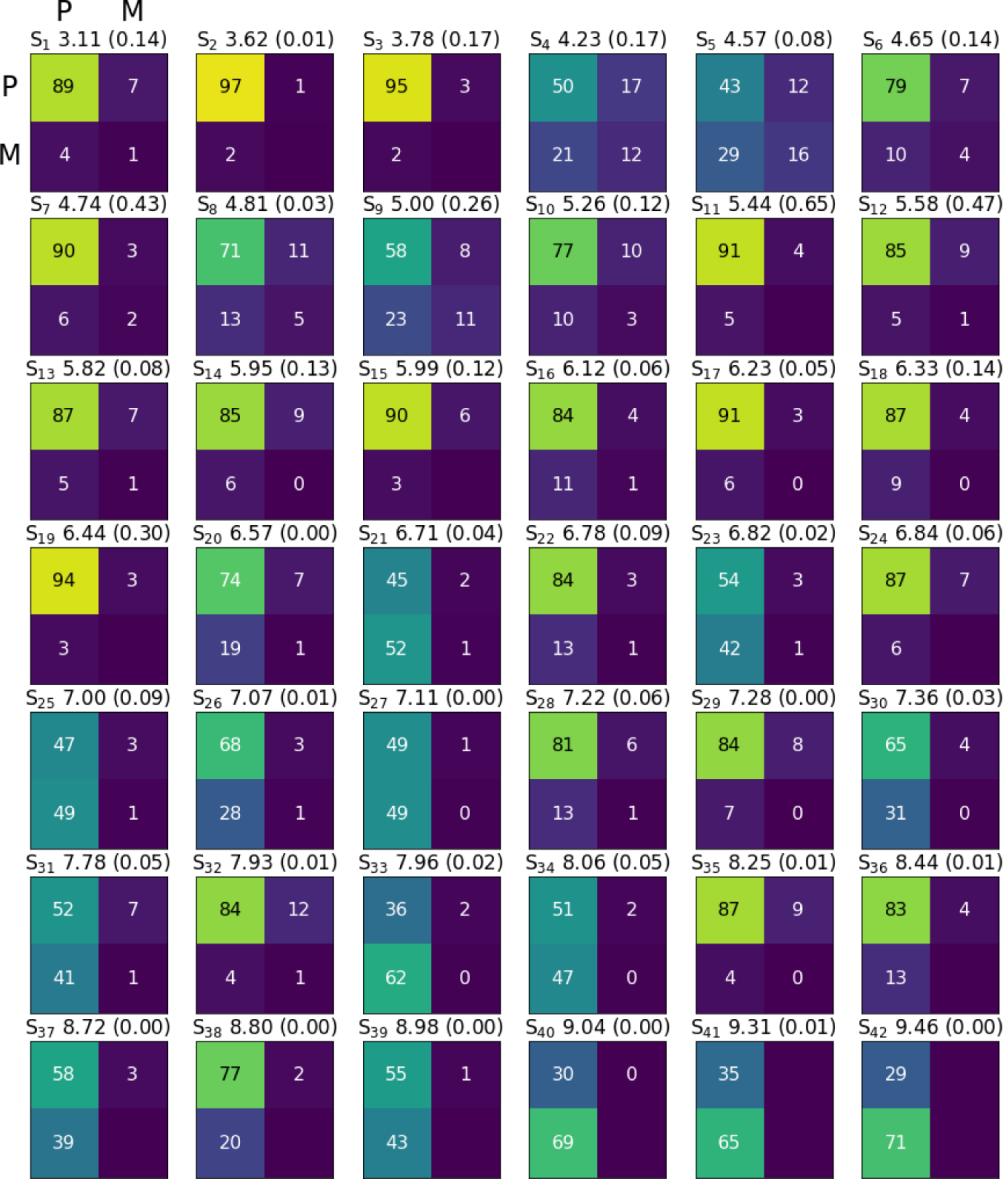
FTDM matrices for the first 42 excited states of the (chemisorption) pyrene–CH_3_Cl­(12-1Cl) complex at the AM1/FOMO–CIS level. P and M letters refer to the pyrene surface and CH_3_Cl molecule, respectively. Notation is the same as that in Figure 4. Here, the “molecule” is defined as the CH_3_ and Cl groups.

We also computed the ground and excited states along the LTP shown in Figure 1e, connecting the pyrene–CH_3_Cl complex with pyrene–CH_3_Cl­(12-1Cl). The results of these calculations are presented in Section S2, where Figure S6 shows the AM1 results and Figure S7 shows the ωB97X-D results. Along the LTP, with both methods, there is an ∼9 eV barrier between the physisorbed reactant and the chemisorbed product in the ground state, *S*_0_. A true transition state will be considerably lower; however, it is still clear that such a reaction cannot proceed thermally. Also, according to the figures along the LTP, the ordering of MS, CT, and other states can change, i.e., crossing potentials and strong nonadiabatic couplings can be expected during NASH dynamics.

### 3.3 Nonadiabatic Surface Hopping (NASH) Dynamics

We modeled NASH dynamics for both the excitation of the noncovalently bound pyrene–CH_3_Cl complex and the excitation of pyrene covalently modified with CH_3_ and Cl (pyrene–CH_3_Cl­(12-1Cl)). We aim to study possible photoreactions between CH_3_Cl and pyrene, as well as potential back-photoreactions. Besides reactions, we are interested in excited-state lifetimes due to intersystem crossing (IC).

#### 3.3.1 Initial Conditions and Spectra from Langevin Dynamics

In the first step, we ran ground-state Langevin dynamics at 300 K for both the physisorbed and chemisorbed systems, as outlined in Section 2.2. As mentioned there, in this way 100 configurations were generated for each case as initial geometries for NASH. As a “side product”, these initial conditions also serve to generate thermally broadened absorption spectra according to the generalized version of eq 2. Including the lowest 21 AM1/FOMO–CIS singlet states *S*_1_ to *S*_21_, the absorption spectra (stick and broadened) shown for both systems in Figure 6a,b were obtained. We see that these spectra largely recover the vertical, nonthermal spectra in Figure 2c,d, respectively, except in the high-energy parts around 8 eV, because in the Langevin-based model less excited states were included.

**Figure 6:**
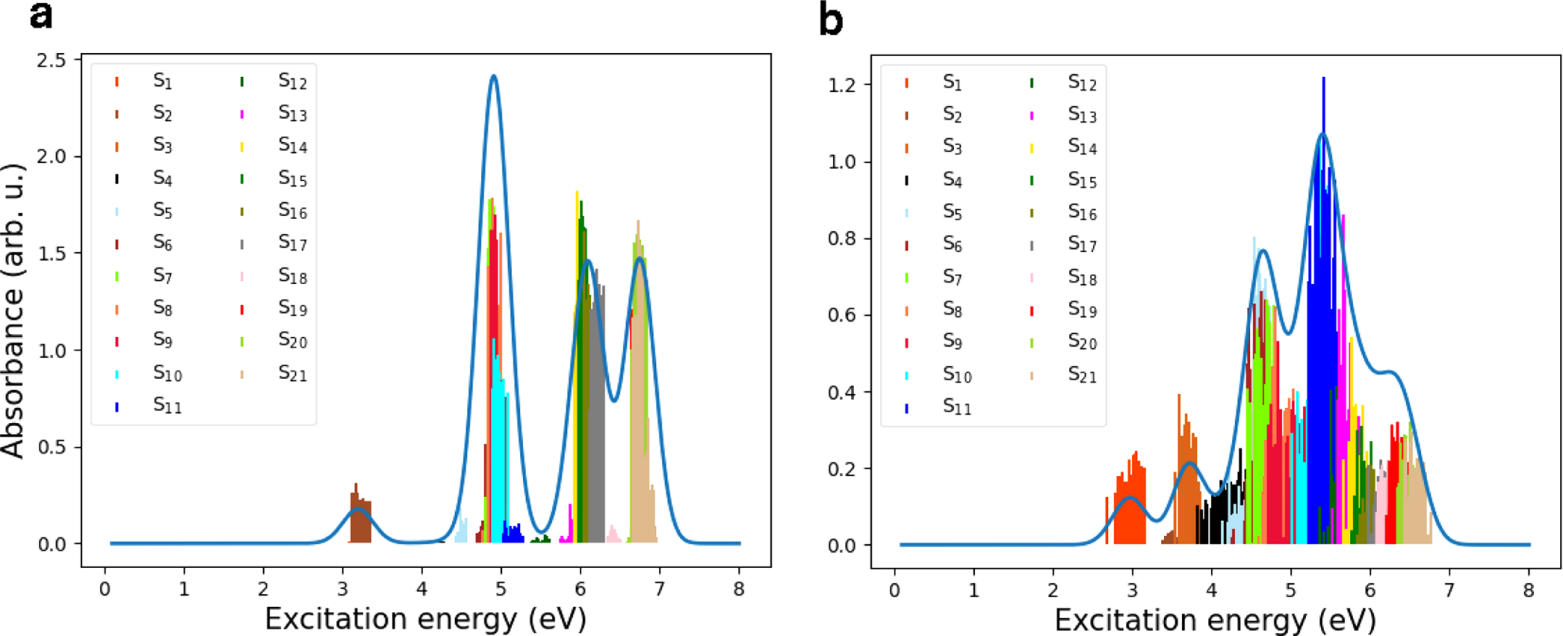
(a) Absorption spectrum (stick and broadened) for the pyrene–CH_3_Cl (physisorption) system calculated at initial geometries selected from the Langevin trajectory, using the lowest 21 excited AM1/FOMO–CIS states. (b) The same as (a), but for pyrene–CH_3_Cl­(12-1Cl) (chemisorption).

#### 3.3.2 NASH Dynamics for Pyrene–CH_3_Cl

The same initial Langevin geometries as those used for the spectra were used for NASH dynamics. Only 11 excited states *S*_1_ to *S*_11_ were considered in NASH, i.e., these were either LE pyrene or MS local states, and no CT or hybrid excited states (which only start at *S*_19_) were included. We used the MS and “mix-of-states” approach (meaning that some trajectories of a swarm were launched in the MS state and others in the LE pyrene state) for initial excitation. Specifically, we performed two separate SH simulations for the pyrene–CH_3_Cl complex: In the first, we used the MS state as the initial state, and in the second, we started from the mix of LE states of CH_3_Cl and pyrene. We note that it would be necessary to include more excited states in the SH simulations to account for the CT excitations (cf. Figure 4). Alternatively, one could attempt to devise a simplified scheme involving a subset of relevant states to address this problem (which, however, goes beyond the scope of the present work).

For the first realization (only MS excitations), initially excited states at a given geometry were selected by inspection and were distributed as follows: 19% in *S*_6_, 11% in *S*_7_, 17% in *S*_9_, 47% in *S*_10_, and 6% in *S*_11_. The mean initial excitation energy amounts to 4.97 eV (∼250 nm). Thus, MS excitation corresponds to the second absorption band of the broadened spectrum in Figure 6a. That being said, we note that oscillator strengths were not considered as a criterion for the selection of initial states. Instead, the initial states were selected by analyzing which orbitals are involved in electronic transitions. The molecular (CH_3_Cl) contribution to the spectrum of the complex, calculated by considering only the states assigned as initial states for the SH simulations, is shown in Figure S8. It is seen there that the extracted CH_3_Cl spectrum has a weak absorption band (with an intensity of ∼0.03) centered around ∼5 eV. While this band is weak, it is more intense than the corresponding band of the isolated CH_3_Cl shown in Figure 2a (with an intensity of ∼0.002).

After running the 100 trajectories according to the protocol outlined in Section 2.3 and analyzing the results, we observed that in 97 trajectories, CH_3_Cl dissociated into CH_3_ and Cl, with no covalent functionalization of pyrene. The dissociation occurs in the excited states. In three trajectories, the CH_3_Cl molecule remained on top of pyrene, continuing to move without dissociating (see Table 2). In this table, quantum yields (Φ) are reported, which are defined as the ratio of *N*_r_ to *N*_t_, where *N*_r_ is either the number of reactive trajectories that result in the dissociation of CH_3_Cl or the number of unreactive trajectories maintaining pyrene–CH_3_Cl without CH_3_Cl dissociation. N_t_ is the total number of trajectories (100 in this case). We note that for the three trajectories where CH_3_Cl remains intact, we observe very rapid surface hops (within the first few femtoseconds) from MS to pyrene states despite small transition probabilities (which is generally possible due to the stochastic nature of surface hopping). Thus, these hops may be artificial to some extent.

**Table 2:** Quantum Yields (Φ) for Reactive (Photodissociation, Leading to Pyrene + CH_3_ + Cl) or Nonreactive Events (Preserving Pyrene + CH_3_Cl) of the Pyrene–CH_3_Cl Complex after Photoexcitation, for the Two Initial State Samplings Described in the Text^a^

excitation	pyrene + CH_3_Cl	pyrene + CH_3_ + Cl	*N*_t_	τ (fs)
MS	0.03	0.97	100	17
Mix-of-states	0.22	0.78	76	60

a *N*_t_, the total number of trajectories, and τ, the “lifetime” of the *S*_6_–*S*_11_ manifold, are also shown.

Further, electronic state populations were computed as fractions of trajectories in the state of interest. To simplify the analysis, we grouped the states as follows: *S*_6_–*S*_11_ (initially excited states), *S*_1_–*S*_5_ (other lower-energy excited states), and *S*_0_ (the ground state). Figure 7a shows the corresponding populations for the pyrene–CH_3_Cl complex for MS excitation.

**Figure 7:**
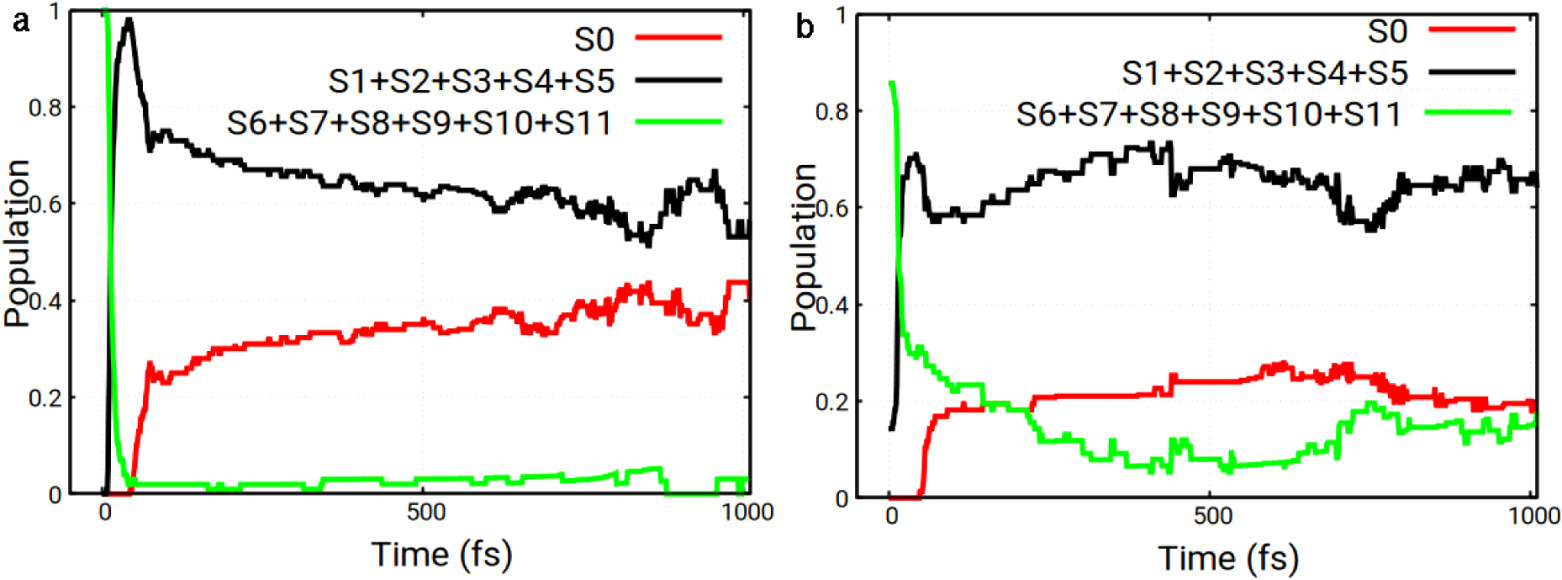
Electronic state populations as a function of time for the pyrene–CH_3_Cl system for MS (a) and mix-of-states (b) initial excitations.

Internal conversion (IC) was observed in all trajectories, with the IC to the group of states *S*_1_-*S*_5_ being extremely fast in all studied trajectories. According to Figure 7a, the decay time is 17 fs when starting from MS, obtained from the monoexponential fit of the green curve:

3 $$P_{S_{6}-S_{11}}\left(t\right)=\exp\left(-t/\tau \right)$$

In the second set of calculations, we studied SH dynamics starting from a mix of CH_3_Cl and pyrene states instead of only MS. This simulation involved 76 successful (nonterminating) trajectories. The mix of states refers to SH with 30 trajectories starting from MS, 27 trajectories from states predominantly associated with pyrene (LE of pyrene), 14 trajectories starting from the MS of CH_3_Cl with some LE contribution from pyrene, and 5 trajectories starting from LE associated with pyrene with a small MS contribution from CH_3_Cl. We should note that some of the selected initial states, particularly several pyrene LE states, are characterized by small oscillator strengths (cf. Figure 4), i.e., effects of small transition dipole moments were not emphasized in this study. The selected initial states were distributed as follows: 1% in *S*_3_, 9% in *S*_4_, 4% in *S*_5_, 12% in *S*_6_, 16% in *S*_7_, 7% in *S*_8_, 30% in *S*_9_, 18% in *S*_10_, and 3% in *S*_11_. The mean initial excitation energy is 4.85 eV, again corresponding to the second absorption band (Figure 6a).

Out of the 76 trajectories, we found that for 59 (78%), CH_3_Cl dissociated, again with no “chemisorption” of either species onto pyrene. In 17 trajectories (22%), the CH_3_Cl molecule remained on top of pyrene, continuing to move without dissociating (see Table 2). Internal conversion was observed in all trajectories: The decay of the population of the *S*_6_-*S*_11_ group occurs with τ = 60 fs in this case (see Figure 7b and eq 3).

In summary, the excitation of only MS or a mix of states leads to qualitatively similar results, namely, dominant CH_3_Cl photodissociation, no pyrene covalent functionalization, and fast internal conversion. Quantitatively, there are differences with the mix-of-states ensemble showing less CH_3_Cl dissociation and longer IC lifetimes.

Figure 8 shows selected distances for 100 trajectories using MS and 76 trajectories using a mix-of-states as a function of time for the pyrene–CH_3_Cl complex when starting from MS (top row) or from the mix-of-states ensemble (bottom row). All trajectories were stable until 1000 fs. Figure 8a shows that the distance between the carbon atom of the CH_3_Cl molecule and carbon atom C2 of pyrene increases, indicating motion of CH_3_Cl (or CH_3_) away from pyrene’s C2 atom. A few trajectories indicate that CH_3_Cl (or CH_3_) remains close to C2; however, no C2–CH_3_ bond is formed. From Figure 8b we see that no C1–Cl bond is formed, while Figure 8c indicates that only a few CH_3_Cl molecules remain undissociated for this initial ensemble. Notably, during the initial phase (time less than 1000 fs), the C–Cl distance remains nearly constant for a few trajectories, suggesting that bond dissociation is time-delayed (by a few tenths or hundreds of femtoseconds) in these cases.

**Figure 8:**
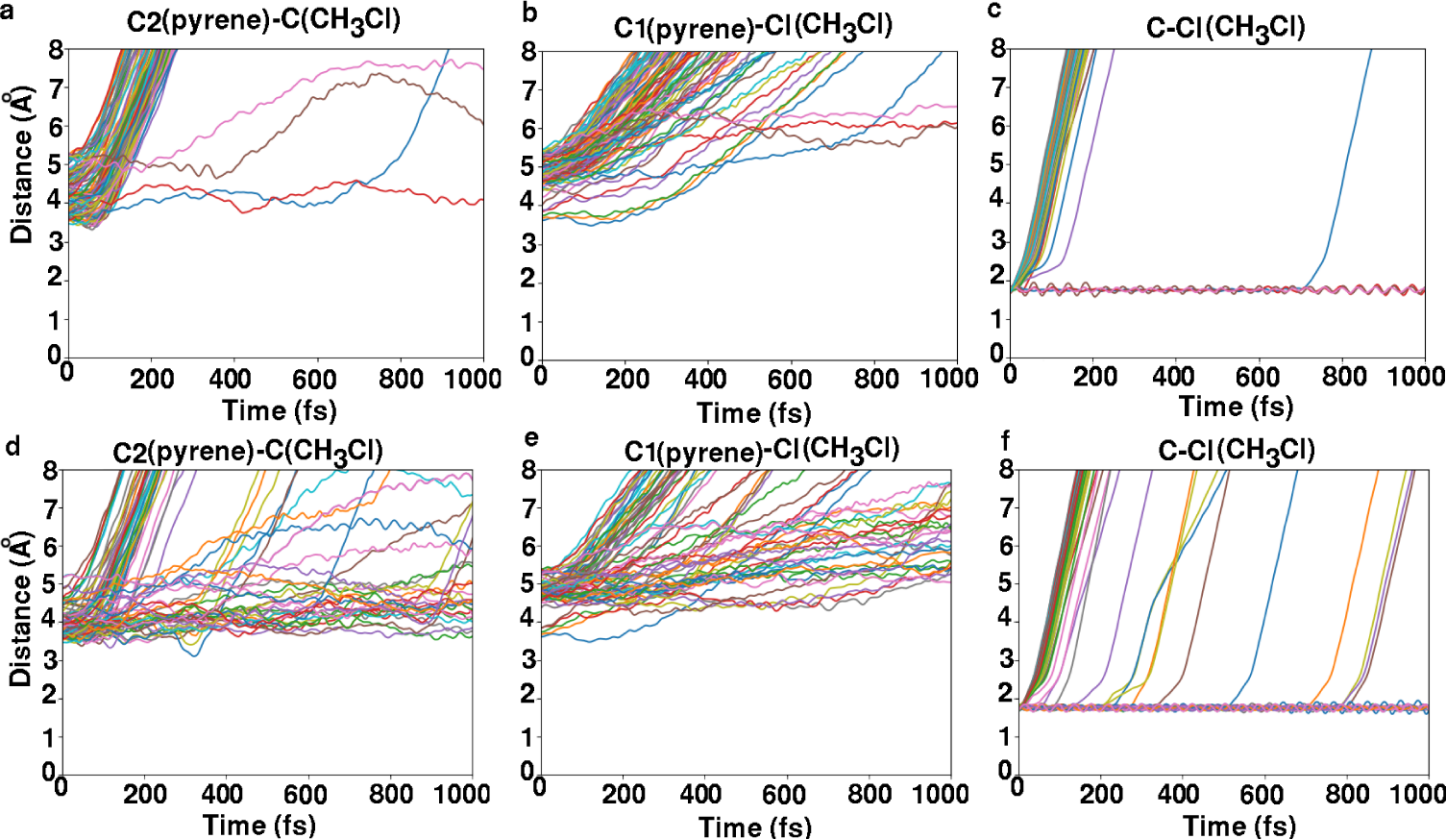
Selected distances for 100 trajectories using MS and 76 trajectories using a mix-of-states as a function of time for the (physisorbed) pyrene–CH_3_Cl system. Panels (a–c) correspond to simulations initiated from the MS state and show different types of interatomic distances (see text), while panels (d–f) show results for simulations starting from the mix-of-states ensemble.

Figure 8d–f shows the same analysis for the mix-of-states initial ensemble. Again, similar behavior of the selected distances is observed as in the case of the MS excitation. However, more trajectories are nonreactive (no dissociation) in the case of the mix-of-states, and more of the dissociation reactions are time-delayed (cf. Figure 8f).

Our results indicate that exciting the LE states associated with pyrene does not facilitate the photodissociation of CH_3_Cl on pyrene, which is not astonishing. What is more astonishing is that already with comparatively low-energy photons (∼5 eV), photodissociation takes place on pyrene after MS excitation, despite the bright states of free CH_3_Cl being much higher in energy, as shown in Figure 2. Further, the fact that neither the MS ensemble nor the mix-of-states ensemble leads to chemical functionalization of pyrene (i.e., the formation of chemisorbed species) has probably also to do with the fact that simply not enough energy is available in our simulation setup to overcome the large barrier toward the formation of this product (cf. the LTP profiles in Figures S6 and S7). That is, higher-energy photons (∼10 eV) and corresponding excited states, including CT states in NASH calculations, are probably needed to observe such a photoreaction. These higher-energy pathways are computationally costly and will be the subject of another investigation.

#### 3.3.3 Surface Hopping for Pyrene–CH_3_Cl­(12-1Cl)

However, even at comparatively low excitation energies, the photochemistry of the chemisorbed species, pyrene–CH_3_Cl­(12-1Cl), is already much richer. In this section, we present NASH dynamics simulations for this complex, starting from two hybrid excited states (H_1_ and H_2_; see Figure 3d for the former). For each case, 100 trajectories were run, each starting from geometries generated from a Langevin trajectory at 300 K.

For the first set of simulations, the H_1_ excitation, the initial states were distributed as follows: 5% in *S*_5_, 3% in *S*_6_, 12% in *S*_7_, 40% in *S*_8_, 38% in *S*_9_, and 2% in *S*_10_. The mean initial excitation energy amounts to 4.96 eV, which is located between the third and fourth absorption band maxima of the spectrum (see Figure 6b).

Compared to SH simulations starting from the pyrene–CH_3_Cl complex, we observe a variety of products, including pyrene–CH_3_Cl­(12-1Cl) (no big structural changes), pyrene–CH_3_Cl* (* indicates that the CH_3_ group is bonded to C2, while the Cl atom is bonded to one of the carbon atoms at the edge, not on the “surface”), pyrene–CH_3_ + Cl, pyrene + CH_3_Cl, and pyrene + CH_2_ + HCl. We find that in one out of 100 trajectories, CH_3_ and Cl were split off pyrene and bonded to form CH_3_Cl and pyrene—a successful “backreaction” (Table 3). In this table, the quantum yields (Φ) for all of the observed channels are provided.

**Table 3:** Quantum Yields (Φ) for Reactive or Nonreactive Events of the Pyrene–CH_3_Cl­(12-1Cl) after Photoexcitation, for the Two Initial State Samplings Described in the Text^a^

excitation	P–CH_3_Cl^b^	P–CH_3_Cl*^c^	P–CH_3_ + Cl	P + CH_3_Cl	P + CH_2_ + HCl	τ (fs)
H_1_	0.02	0.10	0.85	0.01	0.02	163
H_2_	0.04	0.06	0.89	0.0	0.01	192

a τ, the “lifetime” of the *S*_6_–*S*_11_ manifold, is also shown. 100 trajectories were run for each case.

b Pyrene–CH_3_Cl­(12-1Cl) chemisorption complex.

c CH_3_ group is bonded to C2, while the Cl atom is bonded to one of the carbon atoms at the edge, not on pyrene (P).

Internal conversion was observed in all trajectories and is analyzed in Figure 9. According to Figure 9a, the decay time for the *S*_6_–*S*_11_ group is 163 fs (obtained using eq 3).

**Figure 9:**
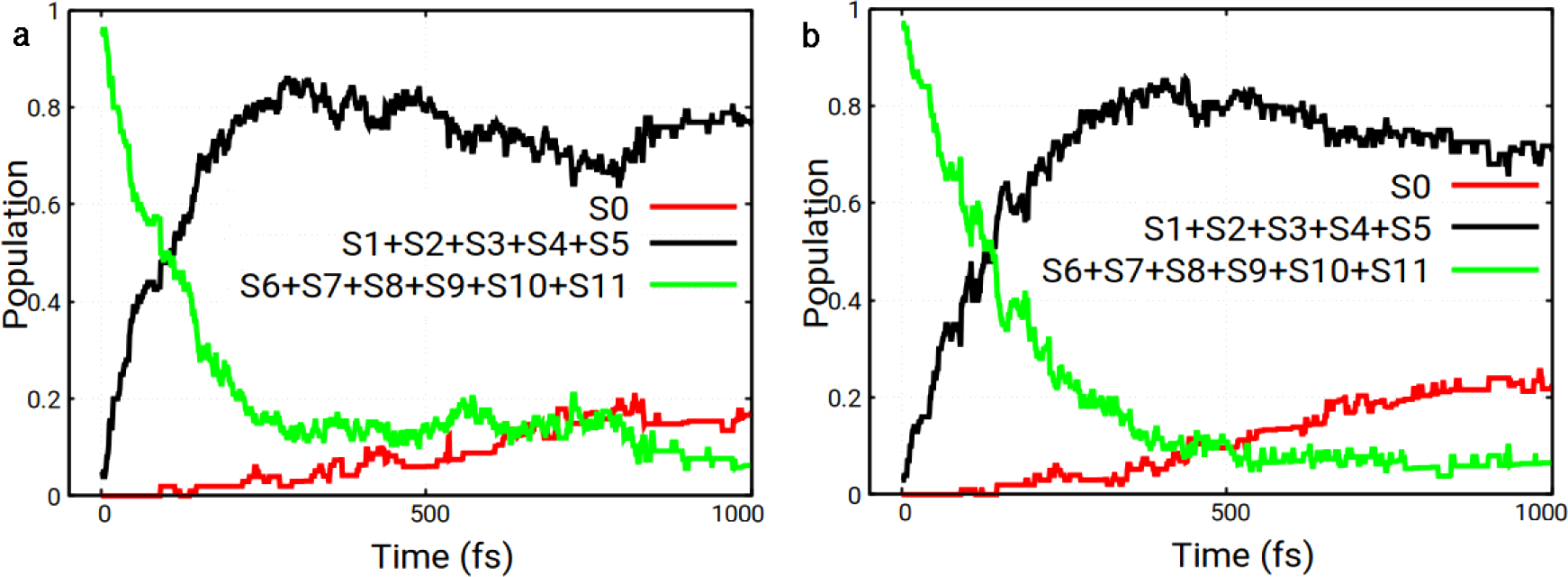
Electronic state populations as a function of time for the pyrene–CH_3_Cl­(12-1Cl) system for H_1_ (a) and H_2_ initial excitations (b).

In the second set of calculations, we studied SH dynamics starting from the hybrid excited state, H_2_. H_2_ and H_1_ states are similar only with different contributions of CH_3_Cl and pyrene. The initial states were distributed as follows: 3% in *S*_5_, 2% in *S*_6_, 7% in *S*_7_, 25% in *S*_8_, 36% in *S*_9_, 22% in *S*_10_, and 5% in *S*_11_. The mean initial excitation energy amounts to 5.06 eV, which again lies between the third and fourth bands of the spectrum (Figure 6b).

We find that out of 100 trajectories each, for 85 after H_1_ excitation and 89 following H_2_ excitation the Cl atom “desorbed from the surface”, leading to the formation of pyrene–CH_3_ + Cl (see Table 3). This indicates that breaking the C1–Cl bond is easier than breaking the C2–CH_3_ bond. Differences between the two excitations, H_1_ and H_2_, are small with respect to reaction yields. Also, population dynamics and lifetimes are overall quite similar for the two initial ensembles under study (cf. Figure 9).

Figure 10 shows selected distances for 100 trajectories as a function of time for pyrene–CH_3_Cl­(12-1Cl), for H_1_ (top row) and H_2_ (bottom row) excitations. Figure 10a illustrates that within the first picosecond the distance between the carbon atom of the CH_3_ group and the carbon atom C2 of the pyrene surface remains unchanged as the simulation progresses. This suggests that the CH_3_ group exhibits no significant tendency to dissociate or migrate away from pyrene at this early stage. In fact, as indicated in Table 3 (for H_1_ excitation), in 97 out of 100 trajectories throughout the entire 10 ps simulation, the CH_3_ group remains bonded to the C2 atom of pyrene. Figure 10b illustrates that the distance between the Cl atom and the carbon atom C1 of pyrene increases along the trajectories, further confirming that Cl is dissociated from pyrene. Figure 10c illustrates the increasing distance between the Cl atom and the carbon atom of the CH_3_ group, signifying the dissociation of the CH_3_Cl “molecule”. Figure 10d–f provides the same analysis for H_2_ excitation, showing very similar behavior to H_1_.

**Figure 10:**
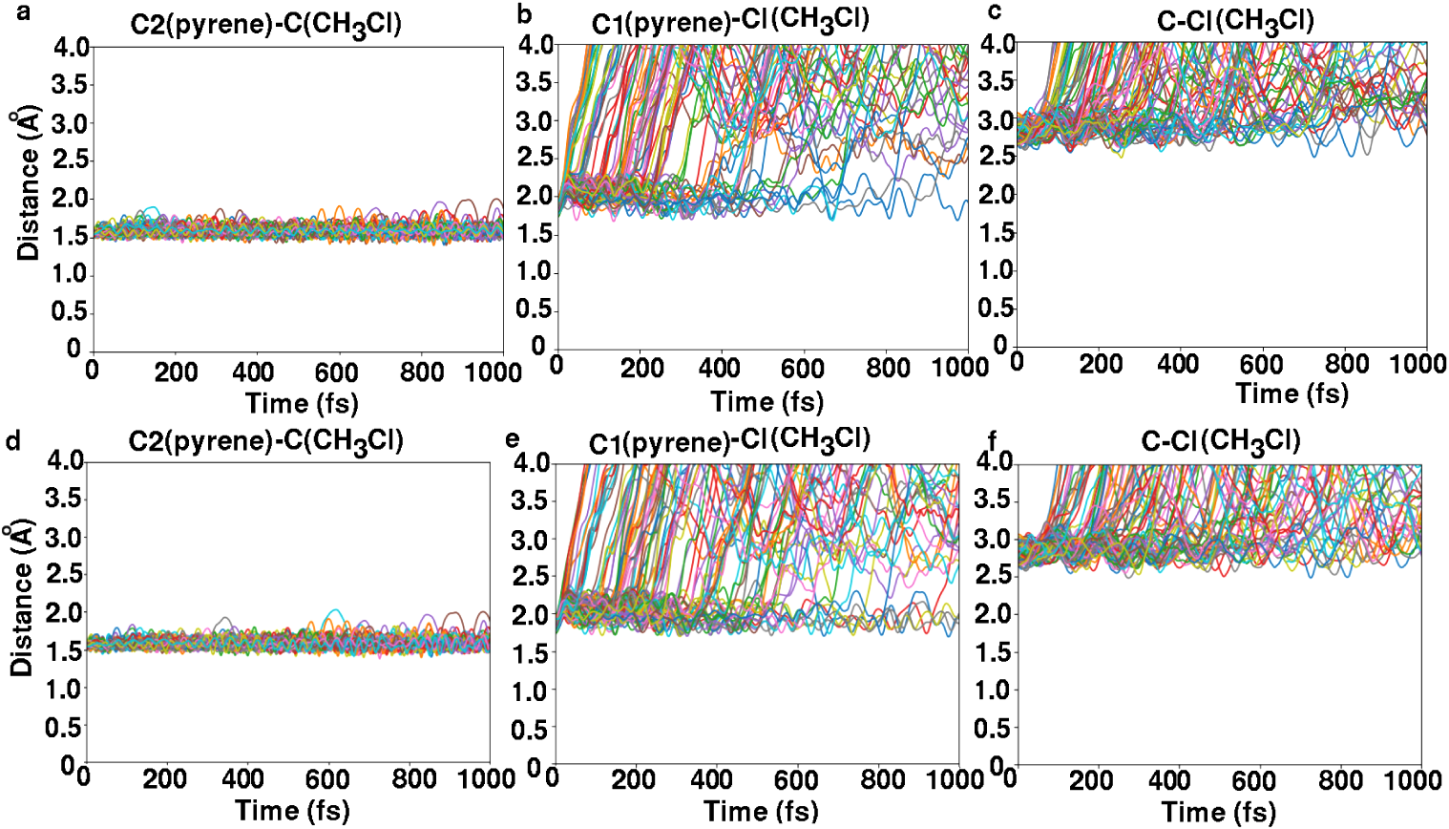
Selected distances for 100 trajectories as a function of time for the pyrene–CH_3_Cl­(12-1Cl) system. Panels (a–c) correspond to simulations initiated in the H_1_ state, while panels (d–f) show results for simulations starting from the H_2_ state.

## 4 Summary and Conclusions

To gain insight into the photophysics and photochemistry of alkyl halides in contact with PAHs, which also serve as molecular models for graphene, we investigated the postphotoexcitation dynamics of pyrene in contact with methyl chloride (CH_3_Cl). We started either from a noncovalent complex or pyrene covalently modified with CH_3_ and Cl. Both first principle methods ((TD-)­DFT) and semiempirical methods (AM1/FOMO–CIS) were used for stationary calculations of ground and excited states, the latter method was also employed for nonadiabatic surface hopping (NASH) dynamics providing direct, atom- and time-resolved insight into these systems after photoexcitation.

The most stable system is the physisorbed pyrene–CH_3_Cl complex, while several possible covalently bound complexes of the two molecules are higher-energy forms. We calculated excited states and spectra and identified key excited states—molecular (MS) and charge transfer (CT) excited states in the case of the noncovalent complex and hybrid states in the case of the selected covalent variant—through natural transition orbitals (NTO) and fraction of transition density matrix (FTDM) analyses. NASH molecular dynamics simulations revealed that dissociation of CH_3_Cl into CH_3_ and Cl is the dominant photoreaction at not-too-high photon energies (around 5 eV), using the pyrene–CH_3_Cl complex as an initial structure. This reaction was observed for 97% of the trajectories starting from MS excited states and 78% starting from a mix of CH_3_Cl and pyrene excited states. The dissociated fragments generally did not covalently functionalize the pyrene “surface” (in contrast to our original hypothesis). As a result, in this case, photoproducts identified by NASH for the pyrene–CH_3_Cl physisorbed complex are primarily pyrene, CH_3_ and Cl. The lifetime due to internal conversion of the electronically excited state manifold was found to be below 100 fs, and in particular, the MS excited states are short-lived. Starting from the “chemisorbed” pyrene–CH_3_Cl­(12-1Cl) complex and applying photons of about the same energies yield a much richer photochemistry and somewhat longer but still comparatively short (160–190 fs) excited state lifetimes. In this case, hybrid states comprising alkyl halide and pyrene orbitals were excited, and among the products, CH_3_Cl (plus pyrene) is formed, but also formation of CH_2_ and HCl has been found and—as a major channel, dissociation of the C–Cl bond.

Overall, this work provides a roadmap for the theoretical treatment of and valuable insights into the photoreactions and excited-state dynamics of pyrene–CH_3_Cl complexes (and other similar (weakly) interacting (halogenated) hydrocarbon complexes), contributing to our understanding of their potential applications to create functional materials or the photodecay of halogenated PAHs, for example. Of course, the consideration of higher-energy (>10 eV) and/or charge-transfer channels (neglected here) is a valuable task for the future, and so is the improvement of the accuracy of the underlying electronic structure methods to make more quantitatively reliable comparisons to and predictions for, experimental results.

## Supplementary Material





## Notes

Coordinates of the structures shown in Figure 1 (XYZ)
Analysis for pyrene–CH_3_Cl complex and pyrene–CH_3_Cl­(12-1Cl) at DFT levels, details about LTP calculations, molecular contribution to the spectrum of the pyrene–CH_3_Cl complex, and potential energy curves for dissociation of CH_3_Cl (PDF)
