# Supplementary material for: Surface Hopping Molecular Dynamics Simulations for Photochemistry Involving Pyrene and CH3Cl
Source: J Phys Chem A. 2025 Jul 24;129(31):7102–14. doi: 10.1021/acs.jpca.5c02583 (PMC12337101; doi:10.1021/acs.jpca.5c02583)
Supplement: Supplementary file 2 [file jp5c02583_si_002.pdf]

Supporting Information for "Surface Hopping Molecular Dynamics Simulations for Photochemistry Involving Pyrene and CH_3Cl"

Elham Mazarei,† Evgenii Titov,† and Peter Saalfrank*,†,‡

†*Universität Potsdam, Institut für Chemie, Karl-Liebknecht-Str. 24-25, D-14476 Potsdam-Golm, Germany*

‡*Universität Potsdam, Institut für Physik und Astronomie, Karl-Liebknecht-Str. 24-25, D-14476 Potsdam-Golm, Germany*

E-mail: peter.saalfrank@uni-potsdam.de

Contents

S1 Analysis for pyrene-CH_3Cl complex and pyrene-CH_3Cl(12-1Cl) at DFT levels **S2**

S2 Details about LTP calculations **S6**

S3 Molecular contribution to the spectrum of the pyrene-CH_3Cl complex **S9**

S4 Potential energy curves for dissociation of CH_3Cl **S10**

S1 Analysis for pyrene-CH_3Cl complex and pyrene-CH_3Cl(12-1Cl) at DFT levels

In this section, we discuss our results obtained with the TD-DFT methods. Fig. S1a, b, and c depict the NTOs representations of the MS and CT excited states for the pyrene-CH_3Cl complex, as well as the hybrid excited states H_1 and H_2 for the pyrene-CH_3Cl(12-1Cl) complex. These calculations were performed at the B3LYP+D3(BJ)/cc-pVDZ level of theory. Additionally, Fig. S2 presents the FTDM matrices for the first 50 excited states of the pyrene-CH_3Cl complex at the same level of theory.

Notably, for the pyrene-CH_3Cl complex, state S_8 (at 5.22 eV) is identified as a CT state (94% M $\rightarrow$ P CT), while state S_{43} (7.30 eV) is an MS state (75% M $\rightarrow$ M LE) according to NTOs and FTDMs obtained at the TD-B3LYP+D3(BJ)/cc-pVDZ level. At the TD-ωB97X-D/cc-pVDZ level, the MS state is S_{26} (7.50 eV) and the CT state is S_{13} (6.51 eV), (see Fig. S3).

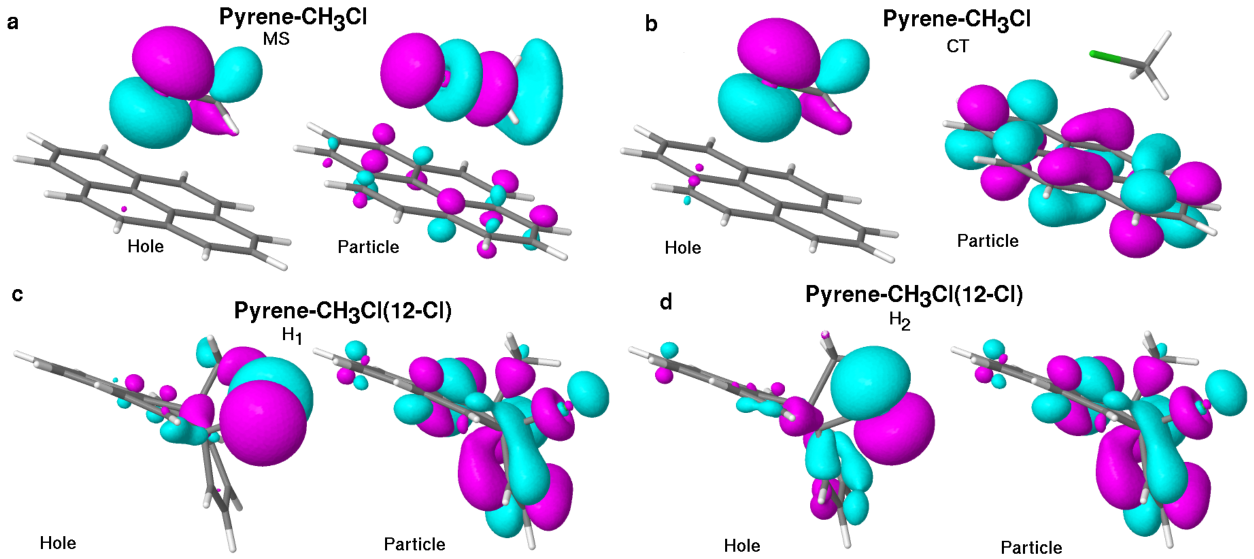

Figure S1: NTOs obtained at the TD-B3LYP+D3(BJ)/cc-pVDZ level: (a) and (b) display MS (S_{43}) and CT (S_8) transitions, respectively, for pyrene-CH_3Cl, (c) and (d) display H_1 (S_8) and H_2 (S_6) transitions, respectively, for pyrene-CH_3Cl(12-1Cl).

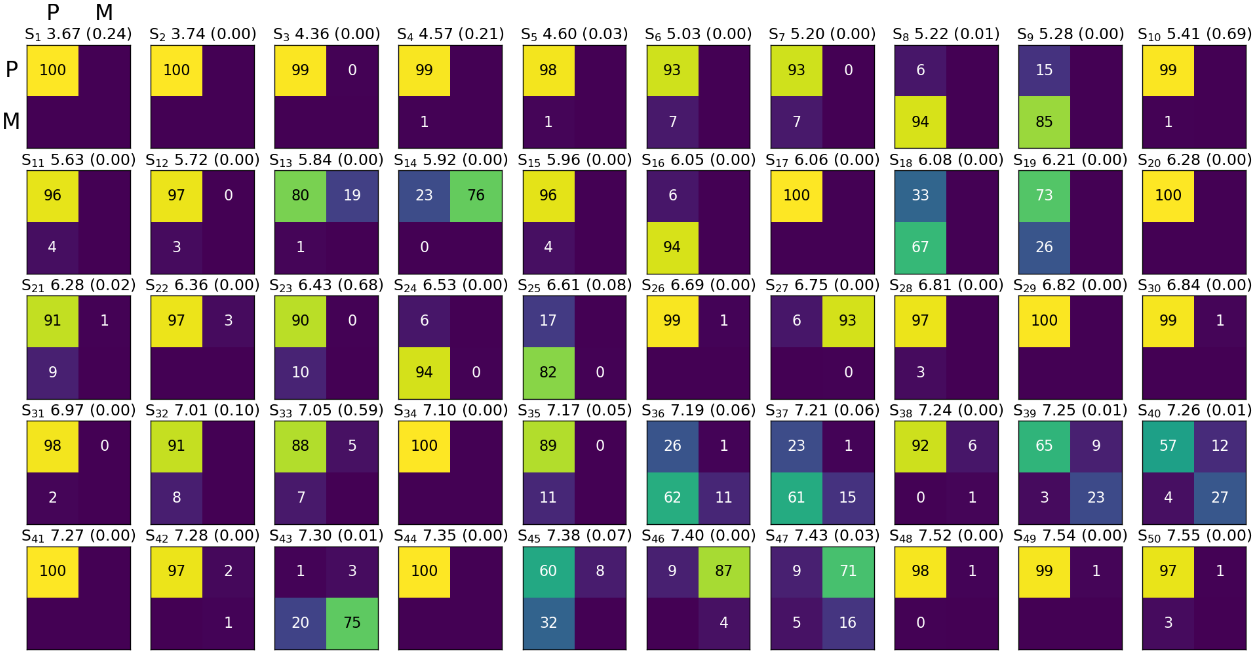

Figure S2: The FTDM matrices for the first 50 excited states of the pyrene-CH_3Cl at the TD-B3LYP+D3(BJ)/cc-pVDZ level. P and M letters refer to pyrene surface and CH_3Cl molecule, respectively. S_1 to S_{50} refer to excited state numbers from 1 to 50, the value after state number is the transition energy in eV, and the value written in parentheses is the oscillator strength of the transition.

Figs. S1c and d display the NTOs representing the hybrid excited states for the pyrene-CH_3Cl(12-1Cl), calculated at the B3LYP+D3(BJ)/cc-pVDZ level. Additionally, Fig. S4 presents FTDMs for the first 50 excited states of the pyrene-CH_3Cl(12-1Cl) at the same level of theory. For pyrene-CH_3Cl(12-1Cl) there are hybrid states. We term the hybrid state S_8 (4.81 eV) H_1, and the hybrid state S_6 (4.66 eV) H_2 at B3LYP+D3(BJ)/cc-pVDZ level. According to Fig. S4, these states are dominated by M $\rightarrow$ P CT ($\sim$65%). At the TD-ωB97X-D/cc-pVDZ level, the H_1 state is S_9 (5.52 eV) and the H_2 state is S_7 (5.32 eV), (see Fig. S5).

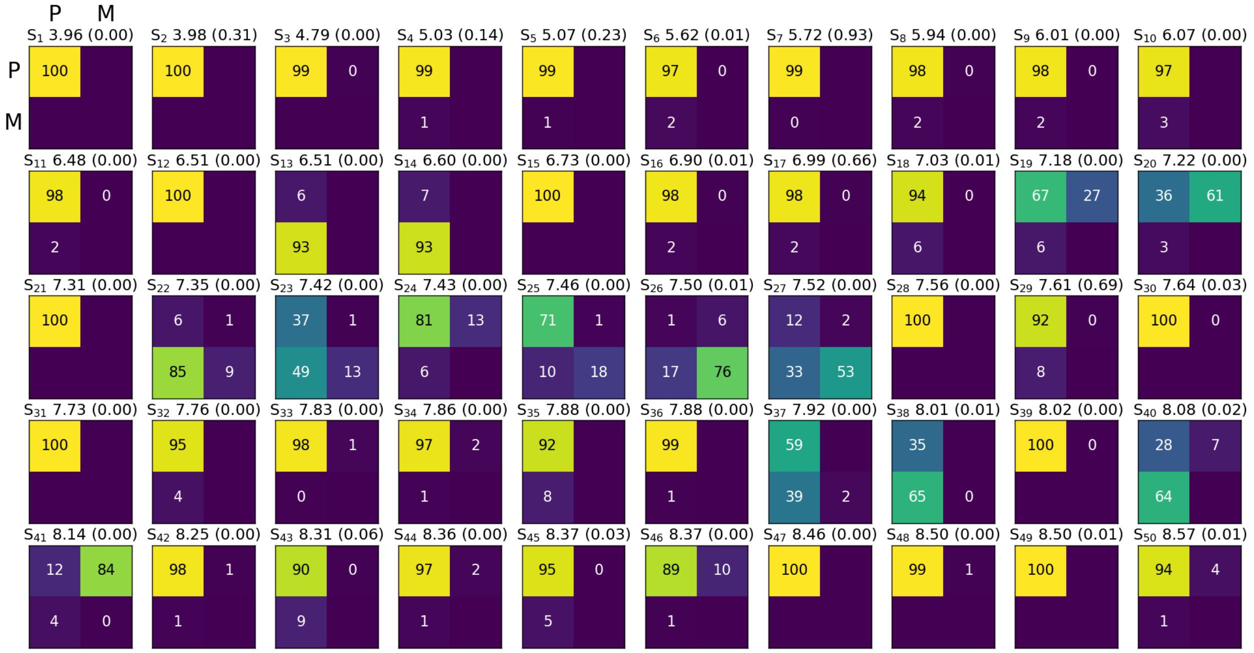

Figure S3: The FTDM matrices for the first 50 excited states of the pyrene-CH_3Cl at the TD-ωB97X-D/cc-pVDZ level. P and M letters refer to pyrene surface and CH_3Cl molecule, respectively. S_1 to S_{50} refer to excited state numbers from 1 to 50, the value after state number is the transition energy in eV, and the value written in parentheses is the oscillator strength of the transition.

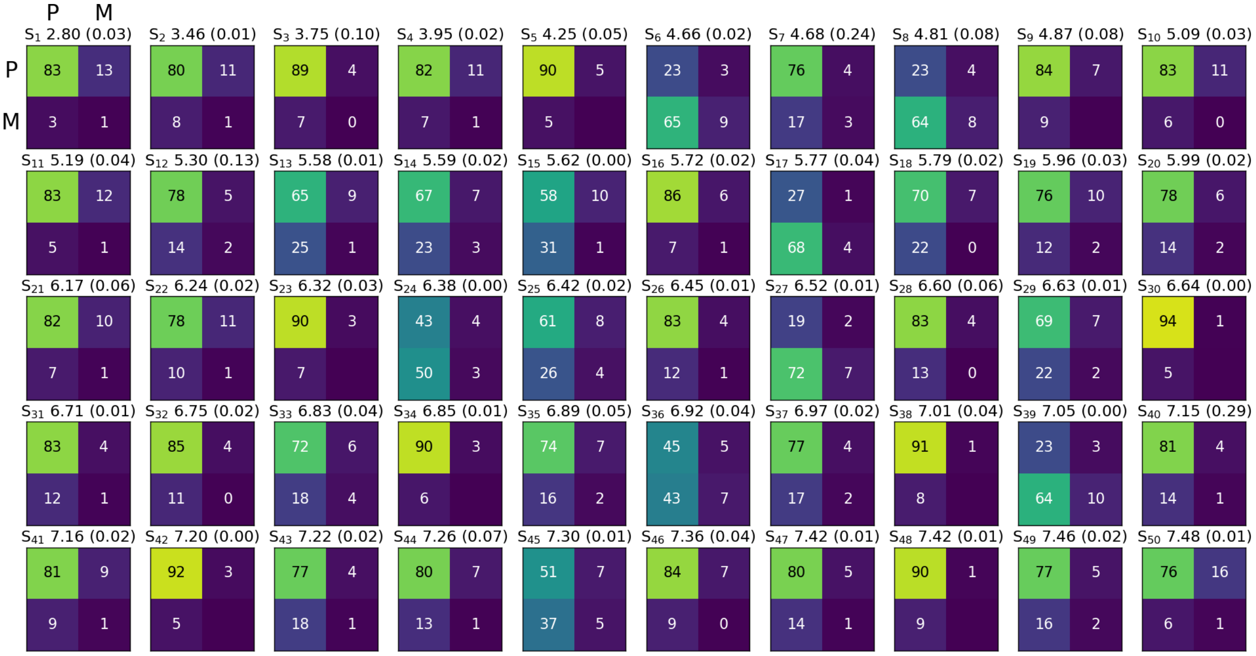

Figure S4: The FTDM matrices for the first 50 excited states of the pyrene-CH_3Cl(12-1Cl) at the TD-B3LYP+D3(BJ)/cc-pVDZ level. P and M letters refer to pyrene surface and CH_3Cl molecule, respectively. S_1 to S_{50} refer to excited state numbers from 1 to 50, the value after state number is the transition energy in eV, and the value written in parentheses is the oscillator strength of the transition.

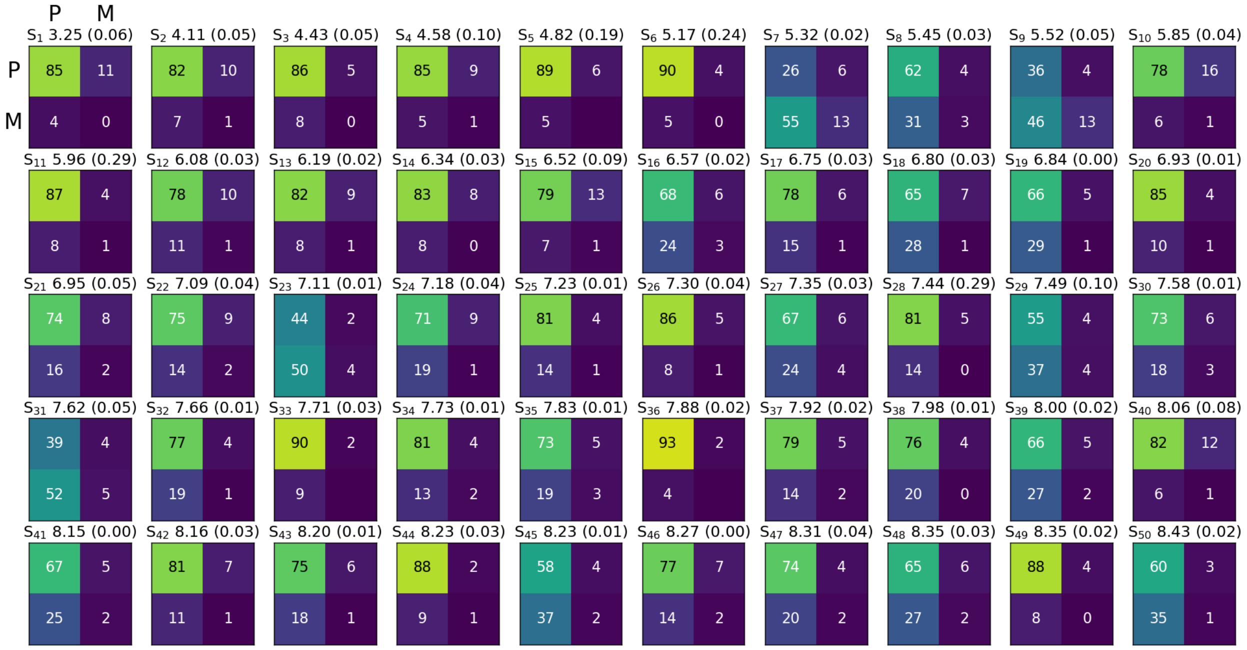

Figure S5: The FTDM matrices for the first 50 excited states of the pyrene-CH_3Cl(12-1Cl) at the TD-ωB97X-D/cc-pVDZ level. P and M letters refer to pyrene surface and CH_3Cl molecule, respectively. S_1 to S_{50} refer to excited state numbers from 1 to 50, the value after state number is the transition energy in eV, and the value written in parentheses is the oscillator strength of the transition.

S2 Details about LTP calculations

We conducted Linear Transit Path (LTP) calculations to elucidate ground and excited-state chemistry of CH_3Cl when in contact with pyrene. This approach allowed us to model the reaction pathway between the non-covalently bound pyrene-CH_3Cl complex and a pyrene molecule modified covalently with CH_3 and Cl, denoted as pyrene-CH_3Cl(12-1Cl). Through the LTP, we illustrate a hypothetical reaction, beginning with the non-covalently bound complex and progressing to the covalently modified pyrene-CH_3Cl(12-1Cl) structure.

In Fig. 1e of the main text, the LTP is shown, while Fig. S7 displays the energies of the first 50 excited states (plus the ground state) for images 0 to 10 of the LTP. Here, the energies (on the y-axis) were calculated as follows:

$$E^j{}_i = E^j{}_0 + \Delta E_i^j - E_0^0 \tag{S1}$$

E_i^j refers to the energy of each state for each image (j labels images from 0 to 10, i denotes the state number from 0 to 50), while ΔE_i^j are excitation energies for image j, and E_0^0 is the ground-state energy of image 0. Furthermore, we analyzed the Natural Transition Orbitals (NTOs) for images 0 to 10 to identify CT and MS states. From image 7 onwards, pure CT or MS states diminish, yielding predominantly hybrid states. We also observe that from images 0 to 6, the CT excitation energy increases, but after image 7, the CT and MS (more precisely, hybrid state) energies decrease.

We note that these non-relaxed LTP calculations reveal large energy differences along the "reaction coordinate": ~10 eV change in the ground-state energy between images 0 and 6, ~6 eV for MS and ~8.5 eV for CT. The large barriers are also observed at the AM1/FOMO-CIS level (Fig. S6).

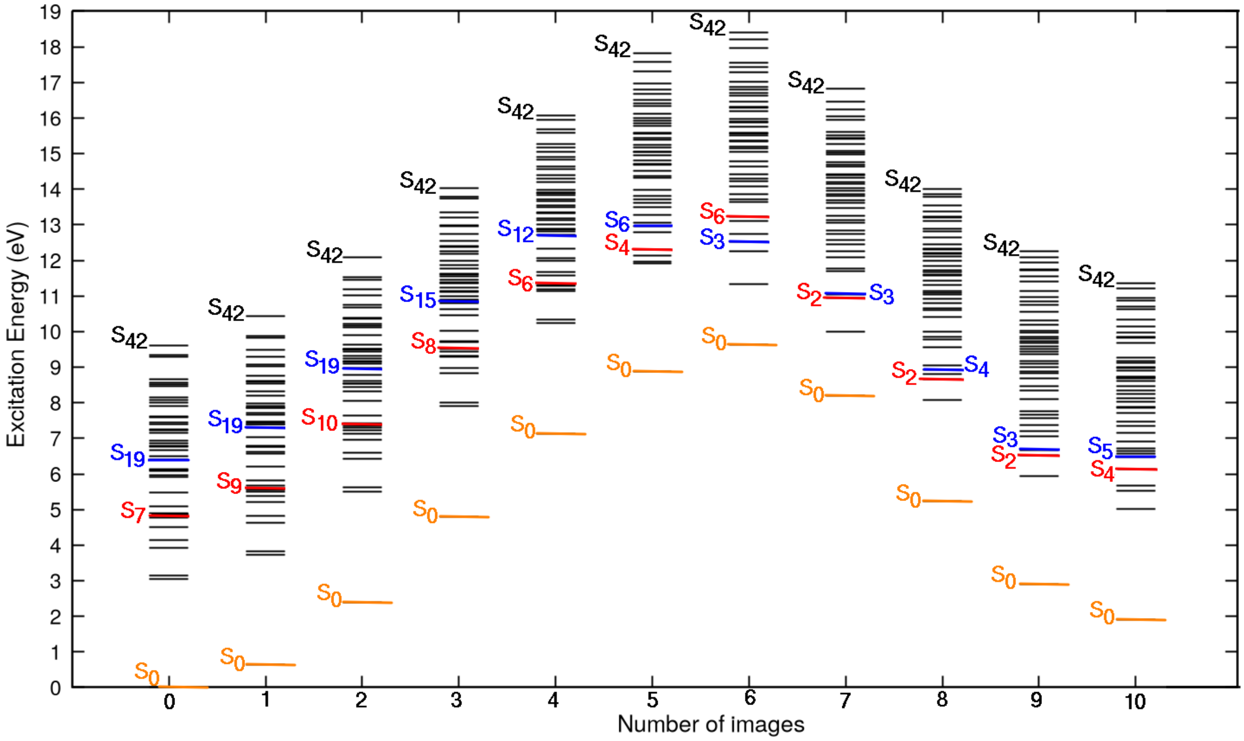

Figure S6: Energy diagram showing the first 42 excited states for images 0 to 10, obtained using the LTP method and calculated at the AM1/FOMO-CIS method. CT states are represented in blue, while MSs are shown in red. S_0 (brown color) shows the ground state for every image.

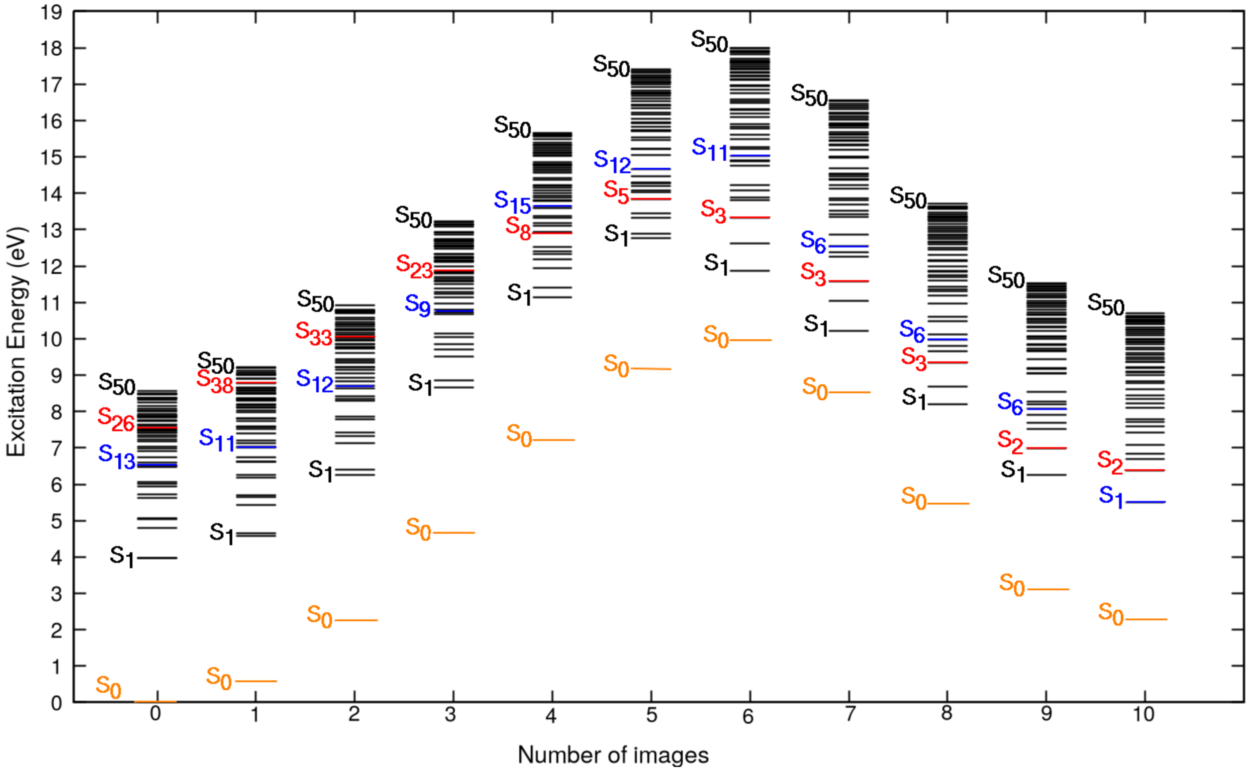

Figure S7: Energy diagram showing the first 50 excited states for images 0 to 10, obtained using the LTP method and calculated at the ωB97X-D/cc-pVDZ level of theory. CT states are represented in blue, while MSs are shown in red. S_0 (brown color) shows the ground state for every image.

S3 Molecular contribution to the spectrum of the pyrene-CH_3Cl complex

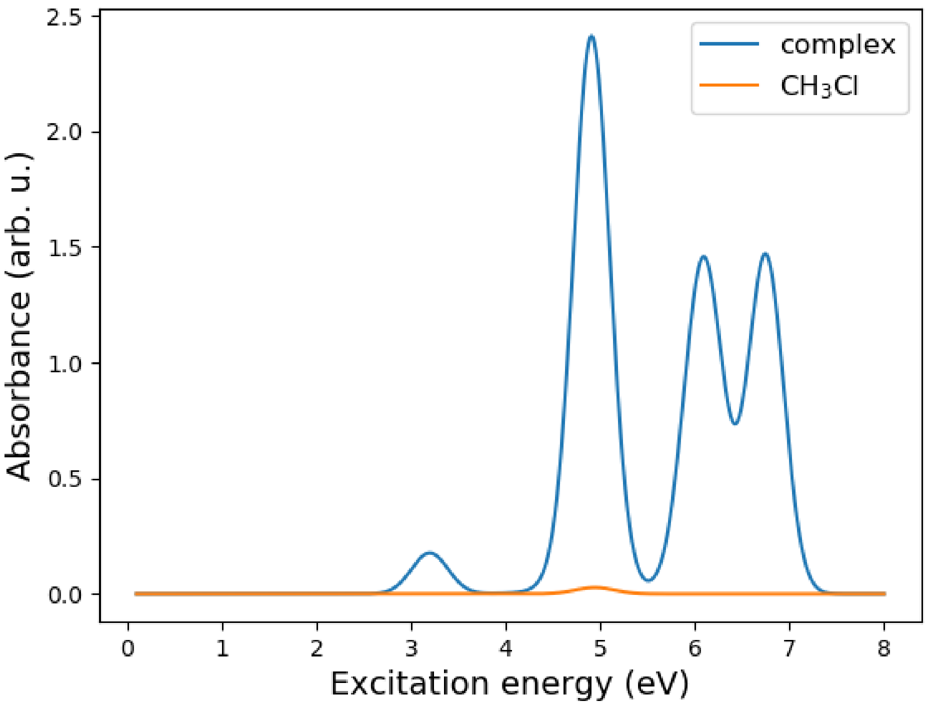

Figure S8: Absorption spectra of the pyrene-CH_3Cl physisorption complex and CH_3Cl extracted from the complex. Calculations were done at the AM1/FOMO-CIS level.

S4 Potential energy curves for dissociation of CH_3Cl

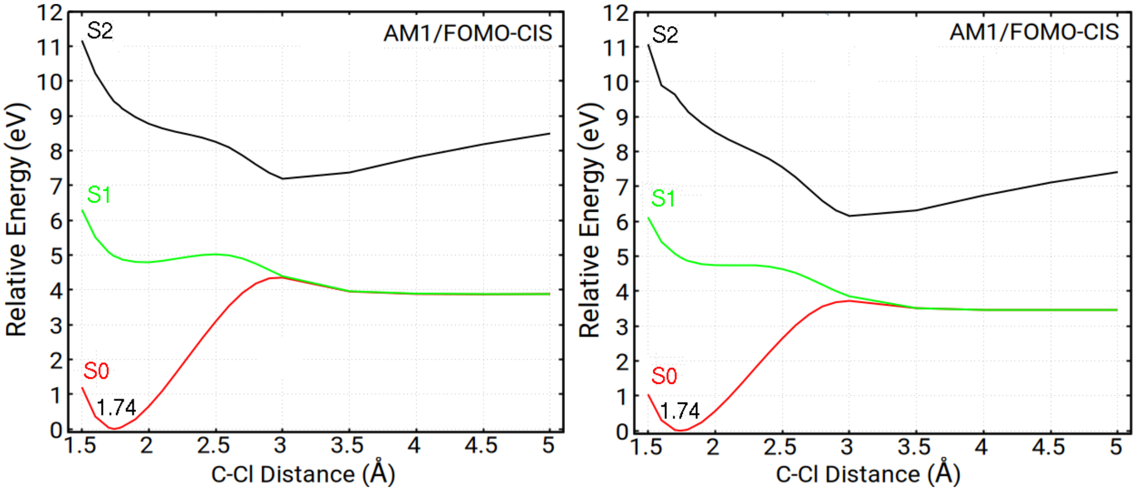

Figure S9: Potential energy curves (left: rigid scan; right: relaxed scan) for the CH_3Cl dissociation along the C–Cl coordinate obtained using the AM1/FOMO-CIS method (with an active space of 6 electrons in 4 orbitals).
